# Postural sensorimotor training versus sham exercise in physiotherapy of patients with chronic non-specific low back pain: An exploratory randomised controlled trial

**DOI:** 10.1371/journal.pone.0193358

**Published:** 2018-03-09

**Authors:** Michael A. McCaskey, Brigitte Wirth, Corina Schuster-Amft, Eling D. de Bruin

**Affiliations:** 1 Department of Health Sciences and Technology, Institute for Human Movement Sciences, ETH Zurich, Zurich, Switzerland; 2 Research Department, Reha Rheinfelden, Rheinfelden, Switzerland; 3 Institute of Rehabilitation and Performance Technology, Bern University of Applied Sciences, Burgdorf, Switzerland; 4 Department of Chiropractic Medicine, University of Zurich, Balgrist University Hospital, Zurich, Switzerland; 5 Department of Neurobiology, Care Sciences and Society, Division of Physiotherapy, Karolinska Institutet, Huddinge, Sweden; Universite de Nantes, FRANCE

## Abstract

Sensorimotor training (SMT) is popularly applied as exercise in rehabilitation settings, particularly for musculoskeletal pain. With insufficient evidence on its effect on pain and function, this exploratory randomised controlled trial investigated the potential effects of SMT in rehabilitation of chronic non-specific low back pain. Two arms received 9x30 minutes physiotherapy with added interventions: The experimental arm received 15 minutes of postural SMT while the comparator arm performed 15 minutes of added sub-effective low-intensity training. A treatment blinded tester assessed outcomes at baseline 2–4 days prior to intervention, pre- and post-intervention, and at 4-week follow-up. Main outcomes were pain and functional status assessed with a 0–100mm visual analogue scale and the Oswestry Disability Questionnaire. Additionally, postural control was analysed using a video-based tracking system and a pressure plate during perturbed stance. Robust, nonparametric multivariate hypothesis testing was performed. 22 patients (11 females, aged 32 to 75 years) with mild to moderate chronic pain and functional limitations were included for analysis (11 per arm). At post-intervention, average values of primary outcomes improved slightly, but not to a clinically relevant or statistically significant extent. At 4-week follow-up, there was a significant improvement by 12 percentage points (pp) on the functional status questionnaire in the SMT-group (95% confidence intervall (CI) = 5.3pp to 17.7pp, *p* < 0.001) but not in the control group (4 pp improvement, CI = 11.8pp to 19.2pp). However, group-by-time interaction effects for functional status (Q = 3.3, 19 p = 0.07) and pain (Q = 0.84, p = 0.51) were non-significant. Secondary kinematic outcomes did not change over time in either of the groups. Despite significant improvement of functional status after SMT, overall findings of this exploratory study suggest that SMT provides no added benefit for pain reduction or functional improvement in patients with moderate chronic non-specific low back pain.

***Trial registration***: ClinicalTrials.gov NCT02304120 and related study protocol, DOI: 10.1186/1471-2474-15-382.

## Introduction

Chronic non-specific low back pain (CNLBP) refers to symptoms associated with pain in the lower region of the back, that have not subsided spontaneously within 12 weeks and cannot be attributed to any specific physiological cause [1]. Up to 85% of individuals presenting for primary care with low back pain (LBP) are said to be non-specific [2]. A cohort study from 2009 found that 42% of all participants presenting with acute LBP go on to develop CNLBP, in 11–12% to a limiting degree in terms of daily activities [1, 3]. The high prevalence of CNLBP in developed countries (approximately 23%) has led to innovative treatment methods and exercise programs [1]. Despite these efforts, CNLBP continues to be the leading cause of years lived with disability, substantially weighing down on health care delivery systems and society [4]. Thus, considering the high socio-economic burden caused by the condition, it remains important to monitor the efficacy of such interventions [5, 6].

The biopsychosocial model [7] has become a popular theory to describe the multifactorial nature of CNLBP [8]. It includes biological aspects of the human movement system (biomechanical and neurophysiological), psychopathological factors (cognitive-behavioural), and their impact on an individual’s social life. While the cognitive-behaviour changes describe how patients with CNLBP may develop fear of potentially painful activities and become over-anxious for their pain [9–11], the biological aspects address the physiological consequences of continued withdrawal from essential physical activity [6, 12]. Both bio- and psychopathological factors are known to result in altered movement behaviour [13, 14]. Particularly the control of posture seems to be affected in patients with CNLBP [15] and sustained aberrant posture could present an often undetected origin for pain initiation or persistence [13, 16–18]. In a review on salutary factors of CNLBP prevention, Rolli-Salathé et al. (2013) describe accurate postural control as a relevant physical resource to protect against the development of CNLBP [19]. Postural control can be defined as coordinated muscle activity to regulate the relationship between the centre of mass (CM) and the base of support during any motor task [20]. Impaired postural control in CNLBP has been described as increased body sway during quiet [21] or perturbed stance [15], delayed lumbar muscle response times to anticipated movements [22] or unanticipated perturbations [23] and rigid trunk strategies in unstable sitting [24]. While these altered movement patterns are often ascribed to isolated muscle atrophy [25, 26], other theories highlight the importance of muscle imbalance [27, 28] due to altered proprioceptive postural control [29]. Paraspinal muscle spindles and Golgi tendons have been shown to be part of the sensory monitoring system that controls the spinal muscles, particularly to provide proprioceptive feedback to the sensory cortex [30]. Corrupted signalling through lesions in this region, intramuscular hematoma and increased intra-compartmental pressure, may impair sensory integrity and lead to prolonged muscle activation related to pain [31–33]. Only recently, the results of a longitudinal 2-year follow-up study have shown that symptom-free participants with reduced proprioceptive postural adaptability were at greater risk (*odds ratio = 3.5*) to develop CNLBP [34] and that reorganisation of specific sensorimotor areas is associated with postural control in patients with CNLBP [35]. This is in line with previous research revealing flawed lumbar position sense (proprioception) in a small cross-sectional study with young patients and controls [36]. Hence, the theory of impaired sensory inputs as a key player in the development and preservation of CNLBP has received considerate attention in recent years [37]. The existence of actual proprioceptive deficits in patients with CNLBP has not been confirmed, but, according to a recent meta-analysis, seems present to some degree in most patients with larger lumbar relative error [38]. Collectively, these findings have given rise to the idea that physiotherapy with a focus on proprioceptive exercises challenging the sensorimotor system can reverse this development, improve local joint control and reduce symptoms in CNLBP [36, 39–41].

The practical application of such sensorimotor training (SMT) in patients with CNLBP involves simple rehabilitation tools like balance boards or elastic bands to elicit neuromuscular provocation and reduce muscular imbalance [39, 41–43]. Within this article, SMT will henceforth be understood as a postural training on labile platforms aiming at the integration and provocation of afferent signalling during standardised training programs and does not include endurance or hypertrophy training [44]. This idea, to use labile surfaces for proprioceptive training, originates from early research on functional instability of the foot by Freeman et al. in 1965 [45]. At the time, the neurophysiological basis of SMT was not fully understood and training effects were expected ‘based upon the hope that some central process might compensate for articular de-afferentiation and its consequent proprioceptive deficit’. It was later suggested that such training methods would ‘enhance proprioception in structures around the knee’ in patients suffering from proprioceptive impairment after anterior cruciate ligament injuries [46, 47]. While these studies failed to deliver convincing evidence of the effects on proprioception itself, as no somatosensory outcome measures were reported, a recent systematic review on the topic concludes that ‘proprioceptive training can yield meaningful improvements in somatosensory and sensorimotor function’ [48]. These effects could not be attributed to SMT specifically, as various forms of active proprioceptive training methods were combined for the analysis. Furthermore, it has not yet been shown whether local proprioception of the lumbar spine can be trained. A Cochrane review evaluated the effects of a similar exercise intervention referred to as motor control exercise [49]. The systematic review concluded that this form of exercise may be more effective than minimal interventions, but not more than other forms of exercise [49]. However, SMT must be differentiated from motor-control exercises, where patients are trained to activate specific deep-layer muscles and reduce overactivation of the superficial trunk muscles while maintaining normal respiration. In contrast, SMT is a global approach to improve proprioceptive acuity in all segments involved in postural movement tasks to reduce muscular imbalances as a potential peripheral source of nociception [39, 41–43].

SMT has become a popular method included in CNLBP rehabilitation, but available reviews on the topic have questioned its biological rational [38, 50, 51] and clinical effectiveness [41]. Only a few studies have been conducted to investigate the effects of SMT on pain and function, suggesting some benefits compared to minimal interventions but not compared to other exercises [41]. However, no study has so far looked into its effectiveness when added to physical therapy, which would represent the clinical application of this intervention. To effectively treat CNLBP, SMT should be part of physiotherapy and should not be applied as isolated exercise [40, 43]. Additionally, although SMT aims to reduce pain and improve function through improved proprioceptive postural control, there is no evidence for its effect on postural outcome measures [41, 52].

As no evidence-based recommendations regarding dose or frequency [52] or for clinical implementation [1] exist, the aims of this study were of exploratory nature. In a small sample, we aimed to evaluate the potential effects of a proposed SMT regime integrated into the usual care of patients with CNLBP compared to sub-effective low-intensity exercise (SLT) with usual care. It was hypothesised that functional status and self-reported pain will change significantly in both groups, but the SMT group would show significantly more improvement when compared to SLT. Using kinematic postural control parameters, this study had the secondary aim to investigate whether SMT improves the multisegmental control of posture, which is a central aspect of SMT. The second hypothesis was that these parameters would improve only in the SMT group.

## Material and methods

### Ethics and reporting

The procedures of this randomised controlled study were first approved by the local ethics committee on November 29th, 2014 (EC North-Western Switzerland, EC number: 2014-337). After study initiation in December 2014, several amendments regarding patient recruitment were made, all of which were approved on April 30th, 2015. Previous versions of the trial protocol limited recruitment to in-house patients only. Due to a low response rate, this was extended to public announcements in local media. Moreover, participants were reimbursed for travel costs and spa vouchers were raffled among all participants. As an additional part of the amendment, patients of the control group were now allowed to choose their preferred endurance exercise (see study intervention description below) and were asked to complete an exercise diary. The provided trial protocol (S1 File) presents the final approved and implemented version and the procedures of all included patients adhered to this latest version (v2.0). The trial has been registered and its protocol published [53]. The current article presents and discusses longitudinal data of primary and secondary outcomes of this trial, except for the proprioceptive outcome of cervical repositioning error. The latter proved to be unfeasible for repeated measures and was omitted from the longitudinal evaluation. The baseline findings are currently being prepared for submission and shall be presented elsewhere. This report follows the Consolidated Standards of Reporting Trials (CONSORT) statement on randomised trials of non-pharmacological treatment [54]. The study conforms to the guidelines of Good Clinical Practice E6 (R1) and the Declaration of Helsinki (2013). No data was recorded before written informed consent was given by the participants.

### Study design

The study was designed as an assessor-blinded exploratory trial with two parallel groups and primary endpoints of pain and functional status. Primary and secondary outcomes were assessed on four measurement events. Baseline data was collected 2–4 days prior to the intervention. Pre-intervention status was assessed immediately before the first treatment and post-intervention within 1–2 days after the last treatment. Additionally, 4 weeks after the last treatment, a follow-up assessment provided data for intermediate-term effects.

### Randomisation, group allocation, and allocation concealment

Randomisation was performed with mixed randomisation steps using block-wise randomisation and simple-randomisation to achieve the unpredictable 1:1 allocation sequence, as has been recommended for smaller group sizes [55]. This was achieved in four steps: First, the sizes of 6 blocks were defined. The size of the first block was randomly set between 5 and 13. Then, three blocks of 4 and two blocks of 6 were shuffled to receive an unpredictable order of block sizes. In the second step, the sequence of group allocation for the first block was randomised with simple randomisation. In the third step, simple randomization with equal group size was applied to the remaining blocks. Finally, the six blocks were merged in the unpredictable block order shuffled in the first step. All steps were executed by a researcher not involved in the assessments or interventions with a one-click macro programmed in Microsoft Excel. Prior to the first treatment, therapists contacted the clinic’s own pharmacy to learn the patient’s group allocation. Blinding of assessors and data analysts was maintained until after study completion. During statistical analysis, the groups were referred to without specification of treatment plan (i.e. group A and B) and only revealed after the final analysis was completed.

### Study population

A convenience sample of 25 patients was recruited at a rehabilitation centre in Switzerland. Upon public announcement, adult patients (≥ 18) with confirmed symptoms of CNLBP presented for baseline assessment [1]. Included patients reported enduring pain symptoms localised primarily below the costal margin and above the inferior gluteal folds for more than 3 months [2]. Patients were excluded if they presented with nerve root pain or specific spinal pathology (e.g. infection, tumour, fracture). Further exclusion criteria were: a history of spinal surgery (e.g. decompression); whiplash incidence within the last 12 months; known vestibular pathologies; inability to follow the procedures of the task. To control for any wash-out effects of previous therapies, patients were only invited when they had not been treated for CNLBP for at least 12 weeks.

### Study intervention

All participating patients were invited to attend 9 sessions of 45 minutes duration consisting of 30 minutes standard physiotherapy according to European guidelines (COST, [1]) with either added experimental (15 minutes SMT) or added control exercise (15 minutes SLT). As no other recommendation exist regarding dosage and frequency of SMT, the choice of the treatment duration was based on transferability to clinical practice, where individual treatments are usually limited to 30 minutes per patient. Although 15 minutes is relatively short in terms of other exercise duration, it was deemed an adequate duration for an intervention that is designed for the implementation in physiotherapy treatments. Sessions were scheduled twice a week over a 4.5-week period at the outpatient department of the trial centre. Five trained PT’s with advanced SMT knowledge (visited at least one certified SMT course) and at least 2 years of clinical experience conducted the treatments. The intensity and duration of SLT in the control group was deliberately instructed to be lower and shorter than is recommended [52]. This was used as a quasi-sham to control non-specific effects of time spent with therapists. Taking part in the study did not affect the patient’s prescribed treatment plan but SMT was not a part of the PT sessions. Other than that, the study protocol did not dictate the PT content or restrict any concomitant care. Details of provided treatments were recorded on therapy documentation sheets and patient diaries. Interventions are described in detail according to the Template for Intervention Description and Replication (TIDieR) guidelines [56] in the published study protocol [53].

#### Experimental SMT group

As mentioned above, there are various methods to apply SMT [37, 41]. For this study, proprioceptive postural training (PPT) with the neuro-orthopaedic therapy device Posturomed™ (Haider Bioswing GmbH, Pullenreuth, Germany) was used. The Posturomed consists of a labile platform, with adjustable damped swaying behaviour. Mediolateral and anteroposterior sway are increased when the two damping brakes, one at the front and one at the back, are released. This allows three specific configurations with increasing levels of instability. In contrast to most proprioceptive training devices, the exercise plan for PPT is clearly defined, quickly explained to the patient and easily understood [57]. Progression of difficulty level and compliance was recorded on a personal exercise diary by the patients and controlled by an exercise therapist at the trial centre.

#### Control SLT group

Patients of the control group received additional sub-effective low-intensity cardiovascular training (i.e. at an inadequate dose to produce effects). Physical activity at low intensity for only 15 minutes is not expected to induce a specific treatment effect to the sensorimotor system [58]. Patients were allowed to choose either the treadmill, elliptical cross-trainer, or a stationary bike and were instructed and positioned according to body constitution by an exercise therapist. Patients were instructed to exercise at a comfortable pace where speaking is still possible (Borg scale 6 to 9) and to maintain this intensity for 15 minutes. Adherence and settings were recorded in their exercise diary after every visit.

### Primary outcomes

#### Functional status

Self-reported impairment in daily activities was assessed using the German version of the Oswestry Disability Index (ODI-G) [59, 60]. The ODI-G is a back-specific, patient-oriented and self-administered questionnaire recommended for the use in studies on the efficacy of interventions for patients with CNLBP [61]. It has shown to be a valid, reliable, and responsive tool to assess to which extent activities of daily living are disrupted by LBP [60, 62]. The ODI-G consists of 10 items addressing personal care, lifting, walking, sitting, standing, sleeping, sex life, social life and travelling. The total score reflects the patient’s current functional status and is reported in percentage of the total achievable 50 points (from 0% = minimal impairment to 100% = bedridden). A change of ≥ 8 percentage points (pp) is interpreted as clinically relevant [63].

#### Self-reported pain

In clinical settings, self-reported pain scales provide an accurate and valid measure of CNLBP severity across time [64]. Current pain intensity was recorded on a 0–100mm Visual Analogue Scale (VAS) with two endpoints representing the extreme states ‘no pain’ and ‘pain as bad as it could be’. The VAS is reported as distance measured on the line between the ‘no pain’ anchor and the patient’s mark on the 100mm line. A change between measurement events of 13mm is interpreted as clinically relevant [63].

### Secondary outcomes

SMT aims to alleviate pain in CNLBP through improved sensorimotor integration, which is believed to reduced muscular imbalance and improved postural response to anticipated perturbations [42]. Therefore, as a secondary analysis, we investigated the effects of SMT on multi-segmental postural control during an experimental postural task.

#### Postural control task

Postural control was assessed on a modified Posturomed with a centrally mounted provocation module that allowed fixation at 3cm deflection in posterior direction. Upon manual release, the platform swayed predominately in anteroposterior direction. Participants were instructed to adopt an upright posture with arms folded across the chest, feet pointed in a natural stance and gaze fixed on a black dot straight ahead. On the cue ‘ready-steady-go’, the assessor released the platform. Two familiarisation trials were performed prior to the measurement. A beep signalled the end of the 10-second measurement. This was repeated five times for every participant. All of the device’s damping brakes were released to allow maximal sway and provoke sufficient postural response. To account for the individual time needed to actively react to the mechanical perturbation as a corrective response [65], an active response phase was derived from the kinematic data [66]. The beginning of the active response phase was defined as first zero-crossing of the CM acceleration after perturbation and ended one second later (see S1 Appendix). The dependent variables (described below) were calculated during this active response phase only, averaged over five trials. Please refer to the S2 Appendix for a detailed description of the measurement setup.

#### Linear measure of centre of pressure displacement: The 95% confidence interval surface area of CP displacement

Postural control is commonly assessed using parameters of postural sway, i.e. the amount of observed CM movement relative to the base of support [20]. An indirect measure which is strongly associated with CM is the trajectory of the centre of pressure (CP) recorded from force- or pressure plates mounted onto the base of support [15, 21, 67]. To assess the amount of sway during the dynamical task, CP oscillation was analysed as CP 95% confidence-ellipse as a measure of magnitude. However, in their systematic review, Mazaheri et al. point out that, ‘increased postural sway may not be present in LBP as consistently as suggested’ and that it should be complemented by dynamic non-linear measures of posture [15].

#### Non-linear measure of centre of pressure displacement: The approximate entropy

Linear measures, like the CEA, quantify the magnitude of CP variation and assume that variance of a time-series is random and are not distinguishable from error. Non-linear measures on the other hand, such as approximate entropy (ApEn), acknowledge that movement variability may also be purposeful to accurately and efficiently perform dynamic movements [68]. In two clinical case studies, healthy states of motor skills have been shown to be associated with an optimal amount of CP variability [69]. Reduced CP variability from this optimal state produces predictable and rigid motor behaviour while excessive amount of CP variability suggests less control of a movement [69]. Clinically, ApEn of CP time-series has shown to detect postural difference in athletes with and without concussion [70, 71] or to predict walking activities based on activity tracker data [72]. Highly predictable time-series are reflected by a lower ApEn value while more chaotic and unpredictable data would be represented by a higher ApEn value [73]. Approximate entropy with dimensionality 2 and a tolerance of 0.2 times the standard deviation was analysed to quantify regularity of the CP time-series [74].

#### Multi-segmental postural control: The uncontrolled manifold index

CP-based measures of postures assume an inversed single-pendulum model with sway predominantly occurring around the ankle [15]. However, SMT is thought to improve the coordinated response of postural muscles around the ankle, hip and neck [42], thus its effectiveness must be assessed along multiple segments. Other studies have also highlighted the importance to assess all major body segments for postural control tasks [75–77]. This poses an analytical problem, as multiple joint-configurations with multiple degrees of freedom allow an infinite number of solutions to achieve a stable posture (i.e. controlling the CM in relation to the base of support). The uncontrolled manifold analysis (UCM) helped to understand how the central nervous system selects a particular combination of joint-configurations from a set of many available configurations. Findings from multiple experimental studies in small samples of healthy participants have shown that rather than controlling the position of each joint, the CNS restricts joint movements that deviate from the task. The variability of joint configurations that lead to the task, i.e. task-specific variability, is high and has been described as a fundamental feature of healthy human movement and motor learning [78, 79]. Task-specific variability is desirable and natural, as it allows compensation of unexpected errors within the movement system [78, 79]. Linear measures obtained from averaged time-series do not capture this variability [76, 77]. UCM analysis is gradually finding its application into pathological movement assessment [66, 80–82]. It has been shown, for instance, that the amount of task-deviating joint variability is greater in fall-prone older people [66] and that the ratio of task-specific and task-deviating variability is lower in children with Down-Syndrom when walking on a treadmill as compared to healthy controls [81]. For the present study, the components of joint angle variability were computed to quantify the amount of variability causing unwanted change (task-deviating) and the amount of variability returning the CM to its steady-state position (task-specific). The relative ratio of both components (UCM-Index, UI) was reported to allow group-wise comparison. Please refer to the S1 and S2 Appendices for the steps required to obtain the variance of these components [77].

### Other outcomes

Socio-demographic data was recorded to characterise the included patients at baseline (age, gender, weight, size, activity level). Activity levels were categorised as inactive (= 1), moderately active (= 2) or trained (= 3) according to minutes spent active per week and based on recommendations of physical activity promotion in primary care [83]. Therapy documentations were analysed qualitatively to compare the type treatments applied during the conventional physiotherapy sessions and to assess adherence to both interventions. Any spontaneously reported unintended effects, discomfort, harms or adverse events were recorded on the case report form.

### Data analysis

Due to non-normal distribution, heteroscedasticity of variance and the small sample size, the parametric tests were deemed inappropriate for this study. Instead, a robust alternative to mixed design general linear model based on 20% trimmed means was conducted to analyse group and time effects [84]. To provide inference about the mean, the lower and upper end of the 0.95 confidence interval for the 20% trimmed mean was calculated. As a robust alternative to Cohen’s d, explanatory measure of effect size was also estimated based on variation among the groups ($\hat{\xi }$). Cohen’s d = 0.2, 0.5, and 0.8 (= small, medium, large effect) roughly correspond to $\hat{\xi }$ = 0.15, 0.35, and 0.50, respectively [84]. In an additional analysis, the overall treatment effect collapsed over both groups was analysed with a repeated measure analysis of variance based on 20% trimmed means. A significance level of post-hoc analysis was adjusted for multiple dependent variables based on the sequential family-wise error correction (Holm method, with *α* = 0.05) [85]. Averaged values for both groups were statistically analysed in R-Studio v. R 3.3.3 running on RStudio (version 1.0.136, 2016, RStudio Inc., Boston) and the statistical package ‘WRS’ v. 30 [84]. Intention-to-treat analysis was performed. Recorded outcome data of patients who dropped out after inclusion and randomisation were included in the final analysis (missing data reconstructed based on carry-forward method). Patients excluded before randomisation were not part of the present analysis.

## Results

**Fig 1:**
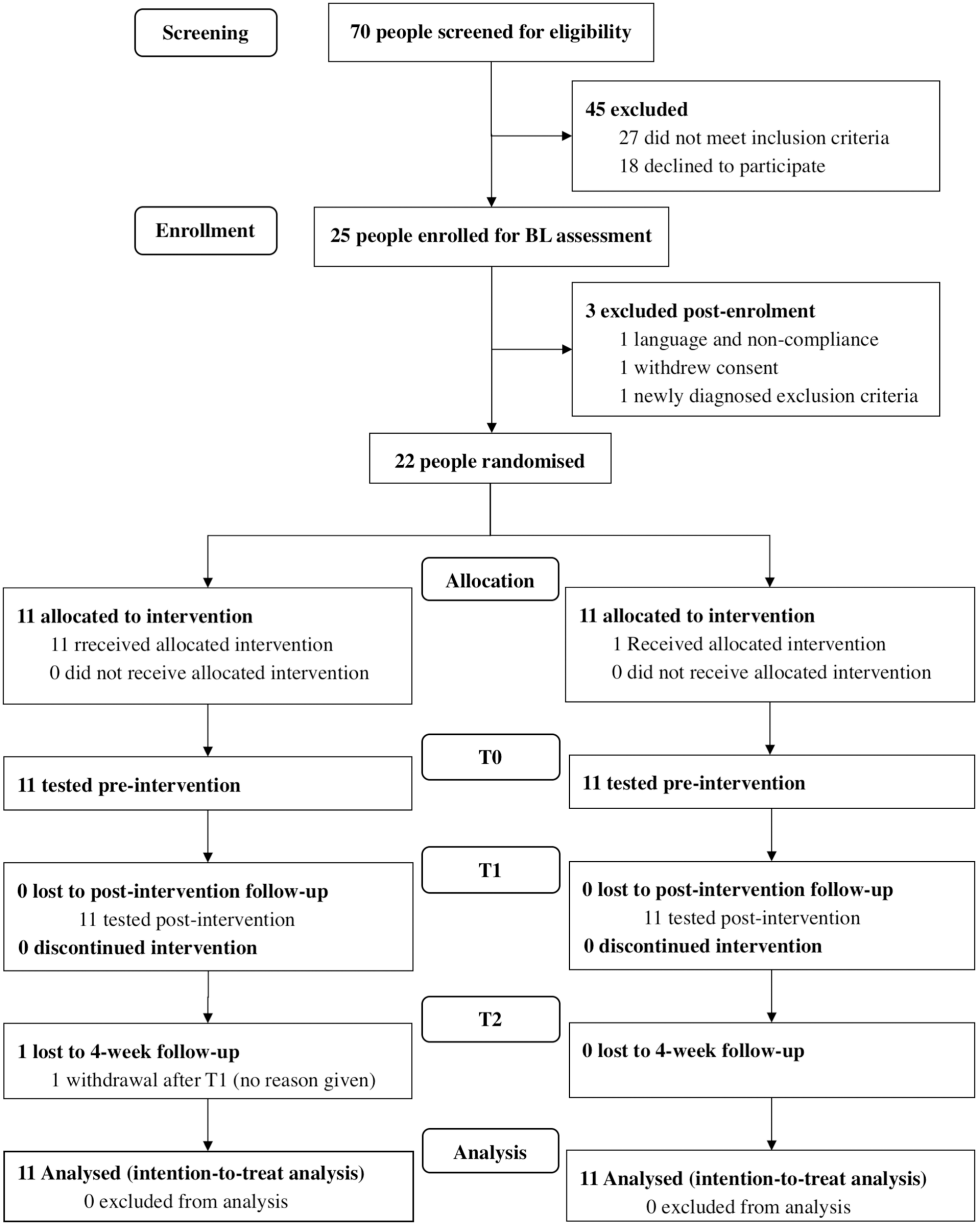
Study flow chart. BL = baseline assessment; *T*_0_ = pre-test, *T*_1_ = post-test, *T*_2_ = 4-week follow-up. SMT = sensorimotor training group; SLT = sub-effective low-intensity endurance training group; PT = standard physiotherapy.

From December 2014 to December 2015, 25 patients were recruited for the intervention trial. Due to the previously mentioned low response-rate, the first patient was not included until June 2015. The last follow-up assessment was recorded in January 2016. Following baseline assessment, three patients dropped out prior to randomization and were not included in the analyses (Fig 1): One patient withdrew due to time restrictions, one patient had to be excluded due to language and compliance difficulties, and one patient was excluded after baseline because a closer clinical examination after referral revealed signs of nerve root compression with sensorimotor deficits in lower extremities. Thus, 22 patients (11 per arm) were included for intention-to-treat analysis (Table 1). One patient in the experimental intervention group failed to appear to baseline and to the 4-week follow-up measurements, but completed all the therapies and immediate pre- and post-intervention tests. In four cases, patients only attended 8 of the 9 therapies due to sickness not related to the study. Hence, 81.1% of patients attended all regular PT sessions. In 72.7% (N = 16), all documentation sheets were flawlessly filed and therapies conducted according to protocol. In one case of the SMT group, the patient failed to attend the additional therapy once. In two cases of the SLT control group, patients failed to attend the additional therapy once. Treatment was not modified during the study and no particular change in lifestyle and activity levels were reported by the patients. Analyses of therapy documentations showed comparable doses and frequency of active and passive treatments in both groups (i.e. mobilisation, strengthening, and passive manual therapy).

**Table 1:** Mean, standard deviation and range values for characteristics of the study population.

	Units	Experimental group (N = 11)	Control group (N = 11)
Gender	f/m	6/5	5/6
Age (range)	years	55 (32–75)	54 (33–67)
Height (SD)	cm	172.4 (11.1)	172.8 (7.9)
Weight (SD)	kg	71.8 (10.5)	72.2 (12.7)
Activity level	1/2/3*	4/4/3	3/6/2
VAS at BL (SD)	%	23.9 (7.1)	25.9 (22.8)
ODI at BL (SD)	%	19.8 (5.3)	17.6 (10.5)

*PAPRICA defined activity levels (1 = inactive, 2 = moderately active, 3 = trained); VAS = visual analogue scale for pain; ODI = Oswestry Disability Index; BL = Baseline.

### Primary outcome measures

#### Functional status

The analysis of the ODI scores revealed no significant group and time interaction (*Q*_*interaction*_ = 3.30, *p* = 0.07). After 4.5 weeks, the average reduction in the SMT group was 6.6 pp (*CI*_95%_ = −6.7*pp* *to* 19.8*pp*) and 5.1pp (*CI*_95%_ = −10.1*pp* *to* 20.8*pp*) in the control group. Both groups improved from baseline to four-weeks follow-up (see Table 2), but only the SMT-group to a significant extent with a within change of 11.5pp (*CI*_95%_ = 5.3*pp* *to* 17.7*pp*, *t* = 7.19, *p* < 0.001). The explanatory effect size for the group effect was $\hat{\xi }=0.11$ and for time effects from baseline to post-intervention and 4-week follow-up $\hat{\xi }=0.45$ and $\hat{\xi }=0.62$, respectively. Interaction effects from baseline to post-intervention and 4-week follow-up were $\hat{\xi }=0.16$ and $\hat{\xi }=0.45$, respectively. Clinical relevant improvement from baseline to post-treatment (≥ 8*pp*) was observed in eight participants, two in the SLT- and six in the SMT-group. At four-week follow-up, 10 patients of the SMT group reported clinical relevant improvement since baseline compared to only two in the control group. The separate analysis of the ODI scores collapsed across groups revealed a significant improvement from baseline to post-treatment and 4-week follow-up (*F*_*t*_(2.4; 31.7) = 6.5, *p* < 0.01).

**Table 2:** Main results of primary and secondary outcomes at 4 measurement events.

	Control T-Mean (*CI*_*L*_—*CI*_*U*_)	Experimental T-Mean (*CI*_*L*_—*CI*_*U*_)	*δ* (*CI*_*L*_—*CIU*)	p
Primary outcomes				
ODI [%]				
BL	16 (4.8–27.2)	20 (14.4–25.0)	4 (−11.5–18.9)	0.5
T0	18 (4.1–31.3)	16 (8.9–23.7)	2 (−20.1–17.2)	0.8
T1	11 (5.7–16.0)	13 (4.9–21.4)	2 (−9.2–13.8)	0.6
T2	12 (7.1–17.5)	8 (2.3–14.2)*	4 (−13.1–5.0)	0.2
VAS				
[mm]				
BL	20.0 (12.0–27.5)	25.0 (17.0–32.5)	5.0 (−7.5–17.5)	0.3
T0	22.0 (9.5–35.0)	25.0 (18.5–31.5)	3.0 (−14.5–20.0)	0.6
T1	13.5 (4.0–23.5)	19.0 (6.5–31.5)	5.5 (−13.5–23.5)	0.4
T2	15.5 (9.0–22.0)	15.5 (3.5–28.0)	0.0 (−16.5–17.0)	1.0
Secondary outcomes				
UI [ratio]				
BL	0.53 (0.42–0.64)	0.46 (0.14–0.78)	−0.07 (−0.50–0.36)	0.62
T0	0.38 (0.08–0.69)	0.41 (0.09–0.72)	0.02 (−0.48–0.53)	0.89
T1	0.51 (0.22–0.80)	0.55 (0.26–0.85)	0.04 (−0.43–0.52)	0.79
FU	0.43 (0.29–0.57)	0.47 (0.12–0.82)	0.04 (−0.44–0.51)	0.80
CEA [cm]				
BL	5.12 (3.04–7.20)	6.79 (4.80–8.79)	1.68 (−1.63–4.98)	0.17
T0	2.34 (0.68–4.00)	3.59 (0.74–6.45)	1.25 (−2.68–5.19)	0.36
T1	3.26 (1.71–4.81)	4.66 (1.47–7.86)	1.40 (−2.93–5.73)	0.35
FU	2.34 (1.64–3.03)	4.80 (1.92–7.67)	2.46 (−1.39–6.31)	0.08
ApEn [ratio]				
BL	0.30 (0.24–0.37)	0.29 (0.26–0.32)	0.01 (−0.10–0.08)	0.69
T0	0.28 (0.22–0.34)	0.24 (0.16–0.31)	0.05 (−0.16–0.06)	0.23
T1	0.28 (0.24–0.32)	0.27 (0.23–0.31)	0.01 (−0.08–0.05)	0.60
FU	0.27 (0.22–0.33)	0.21 (0.17–0.26)	0.06 (−0.14–0.03)	0.07

Trimmed means (T-mean, 20%) of primary and secondary outcomes at baseline (BL), pre- and post intervention (T0 and T1), and 4-week follow-up (T2). *δ* = mean difference between groups, *CI*_*L*_ and *CI*_*U*_ = upper and lower 95% confidence interval, p = p-value of between-difference.

*Significant within-change since BL (*p* < 0.001).

#### Self-reported pain

At four-week follow-up, eight participants improved by more than 13mm, four in each group. From baseline to post-treatment, VAS-pain scores decreased by 6.6mm (*CI*_95%_ = −5.0*mm* *to* 18.2*mm*) in the control group and by 5.6mm (*CI*_95%_ = −16.8*mm* *to* 28.2*mm*) in the SMT group (Table 2). Accordingly, there was no significant group, time or interaction effect (*Q*_*between*_ = 0.63, *p*_*between*_ = 0.44; *Q*_*within*_ = 1.92, *p*_*within*_ = 0.19; *Q*_*interaction*_ = 0.84, *p*_*interaction*_ = 0.51). The explanatory effect size for the group effect was $\hat{\xi }=0.20$ and for time effects from baseline to post-treatment and 4-week follow-up $\hat{\xi }=0.32$ and $\hat{\xi }=0.36$, respectively. Interaction effects from baseline to post-treatment and 4-week follow-up were $\hat{\xi }=0.03$ and $\hat{\xi }=0.14$, respectively. Clinical relevant improvement from baseline to post-treatment (≥ 13*mm*) was observed in nine participants, four in the SLT- and five in the SMT-group. Overall, VAS scores also improved significantly from baseline to post-treatment and 4-week follow-up (*F*_*t*_(2.4; 31.1) = 4.0, *p* < 0.02).

### Secondary outcome measures

The kinematic data of multisegmental variance (UI) showed no change over time suggesting there was no effect of either treatment on dynamic postural strategies (Table 2). Kinetic data of CP confidence ellipse (CEA) also remained unchanged across all trials in both groups. For both groups, signal structure remained unchanged in terms of regularity and predictability (ApEn). The explanatory effect size for the UI group effect was $\hat{\xi }=0.003$. The explanatory time effects from baseline to post-treament and 4-week follow-up were $\hat{\xi }=0.05$ and $\hat{\xi }=0.11$, respectively. The explanatory effect size for the CEA group effect was $\hat{\xi }=0.44$ and for time effects from baseline to post-treatment and 4-week follow-up $\hat{\xi }=0.44$ and $\hat{\xi }=0.58$, respectively. The explanatory effect size for the ApEn group effect was $\hat{\xi }=0.31$. The explanatory time effects from baseline to post-treatment and 4-week were $\hat{\xi }=0.18$ and $\hat{\xi }=0.46$, respectively.

### Safety

All patients tolerated the SMT program and no serious adverse events occurred. All patients rated trial procedures as tolerable, but in one patient, for no explainable reason, clinically relevant worsening of functional status by 10pp on the ODI scale was observed.

## Discussion

This exploratory clinical trial examined the effects of SMT as part of standard physiotherapy programs during CNLBP rehabilitation compared to sub-effective low-intensity endurance exercise. At the end of the treatment series, a substantial reduction in functional impairment and self-reported pain was noted in both groups. Particularly at 4-weeks follow-up, the SMT group had significantly improved since baseline. However, no significant group-by-time effects on either of the primary or secondary parameters were observed, suggesting that, on average, attending additional low doses of SMT units on a regular basis does not have any significant benefits when compared to low levels of added general activity. Both groups showed no change of motor reaction to the perturbation task submitted to the patients during the postural control assessment.

This is the first randomised trial evaluating the effects of SMT with equal group sizes, a standardised and theory-based training program, and a comparable active control group with equal time spent with therapists. Other trials with similar aims have been summarised in a recent systematic review [41]. Although most trials showed promising effects, the low quality of the cumulated findings prevented final conclusions and clear recommendations on which a large-scale study could have been based upon [41]. One trial claims that SMT allows patients to regain muscular balance, which is supposed to be partly responsible for pain alleviation and improved neuromuscular coordination [86]. However, in that particular study doses and frequency were higher than in the present study with five 40 minute treatments per week during four weeks. Moreover, the results were only compared to passive controls. Thus, the observed effects may be non-specific rather than being attributable to SMT alone. Other research in elderly populations has suggested that SMT may be beneficial as part of exercise programs to improve balance and reduce risk of falls, but not to a greater extent than usual exercise [87].

Addressing specificity of SMT, in a narrative review on the topic, Kim et al. (2011) point out that there seems to be no formal definition of what SMT is or what it should entail [58]. When compared to other exercises, both approaches are likely to be equally effective in terms of improved function in CNLBP [41] or for injury prevention and it has been suggested that there might be no such thing as specific sensorimotor exercise [88]. Instead, improvements may be attributed to non-specific effects of exercise and physical activity [88]. In order to evaluate the specific effects of SMT, there must be some degree of standardization for implementation and recommendation [52]. Currently, recommendations regarding the implementation of SMT can only be derived from narrative reviews and expert opinions [42, 50, 58, 89, 90]. According to these, there are three training principles suggested to be of particular relevance if any effect from SMT could be expected: First, the level of instability must be adjustable and incremental over time. The participant must be able to control the task to complete the exercise properly, but still be challenged when progressing his or her skills [40, 58]. Second, the participant must be able to respond to the instability, i.e. there must be a closed-loop control system in which feedback is compared to an intended goal [40, 58]. Finally, the exercise at hand must include a secondary task (i.e. dual task) which is separated from the functional stability task (e.g. juggling a ball or cognitive challenge) once the participant has advanced to a certain level in order to centralise the acquired skills [58]. While the present study is the first to actually adhere to these principles [41], the chosen duration of the present study was arbitrary as no indication regarding dose or frequency was available. Although the doses and frequency chosen were similar to previous studies with active comparator groups, e.g. three times 15 minutes a week for five weeks in a study on the effects on neck pain [91], the relative amount of added experimental therapy might have been too short with the main effect of the treatment owed to the actual treatments applied during standard PT. SMT targets postural muscles vastly consisting of aerobic type I muscle fibres (e.g. erector spinae). These muscles are inherently fatigue resistant and would likely require high volume and frequency to provoke any training effect [90]. The population sample in the present study presented with moderate to low pain levels and generally reported to be leading an active lifestyle. The putative training effect of SMT is likely to be more pronounced in patients with lower levels of activity and higher pain levels and more pronounced functional impairment.

Whether the UCM analysis is an appropriate measure to evaluate movement variations in pain affected people is certainly a point of discussion and should be investigated in more detail, possibly with other movement tasks that have shown to deviate in CNLBP patients. One study has shown reduced task-specific variability during a sit-to-stand task in young CNLBP patients with low pain levels [82] while another study found increased task-specific variability during a postural task on a labile surface, indicating more segmental variation to achieve the same goal [92]. Despite the number of trials produced on the topic, the sensitivity of the UI has not been investigated yet and our trial might have been underpowered to show the expected effects. A previous clinical trials with comparable sample sizes used UI to show pre- to post intervention changes in gait, but the population under investigation (Down Syndrome) was less heterogene and had more evident postural deficiencies [81]. The wide age-range of the included patients may have further masked the effect of the treatment on secondary outcomes as it is well established that balance is affected by ageing [66, 93]. Yet, restricting the upper limit of age would not have represented a clinically relevant population for the primary outcomes and would impair the accuracy of any estimates for larger studies on the topic [94]. Moreover, it has also been shown that the effect of age on the analysed outcomes is only significant in populations older than 70 [66, 93].

Despite attempts of blinding the patients to the experimental condition through a quasi-sham intervention, the intervention could not be entirely blinded to the participants, for which reason non-specific effects contributing to the minor differences observed cannot be ruled out. Further, leisure activities of the patients could not be controlled or restricted during the study. However, no particular change in lifestyle habits was reported from any patients.

The presented exploratory study shows a positive trend with high interactions effect sizes for the improvement of functional status in CNLBP after receiving added SMT in standard physiotherapy programs, albeit without any change in postural control. Our findings provide a basis for further intervention trials testing the effectiveness of SMT, but the small sample size is insufficient to demonstrate clinically relevant results. Thus, they should be interpreted with caution and must be confirmed with further studies [95]. Only in combination with more methodologically comparable studies and a quantitative meta-analysis can these findings lead to clinically relevant recommendations [96]. Such studies should comply with international guidelines for randomised controlled trials [54]. They should describe SMT in detail [56] and include a minimum of standard parameters of postural control to investigate its role in SMT and CNLBP recovery. Moreover, to provide evidence of the trainability of the sensorimotor system through balance training, it must be shown that acuity of sensory receptors and the signal conversion to and within the CNS can be enhanced [58]. It is yet to be defined how much SMT would be necessary to induce any short- and long-term effects from SMT. Based on our results we recommend higher doses than have been described in the present study, which implies that it must be part of additional home exercise as the available time spent with physiotherapists is limited.

## Conclusion

In patients with moderate chronic non-specific low back pain, physiotherapy with added sensorimotor training or sub-effective low-intensity training improved impaired functioning with no significant group difference. Short-term effects on pain and function seem to be similar for either kind of added activity, but the findings suggest potential benefits of SMT for long-term functional status. No improvement in terms of postural response to platform perturbation was observed. Multi-segmental postural control and centre of pressure remained unchanged throughout the trial.

## Supporting information

S1 Appendix Kinematic equations. (PDF)

S2 Appendix Measurement setup. (PDF)

S1 File SeMoPoP Study protocol approved by ethical committee. (PDF)

S2 File CONSORT checklist. (PDF)
