# Supplementary material for: Postural sensorimotor training versus sham exercise in physiotherapy of patients with chronic non-specific low back pain: An exploratory randomised controlled trial
Source: PLoS One. 2018 Mar 9;13(3):e0193358. doi: 10.1371/journal.pone.0193358 (PMC5844549; doi:10.1371/journal.pone.0193358)
Supplement: S1 Appendix — (PDF) [file pone.0193358.s001.pdf]

S1 Appendix: Kinematic equations

Joint angles and center of mass excursion.

The sagittal marker coordinates were used to calculate the joint angles of the foot (θ_F), ankle (θ_A), knee (θ_K), hip (θ_H), lumbar (θ_L) and neck (θ_N) [1]. Based on estimated segmental CM and mass proportions, weighted sagittal plane CM location was computed for every frame [1]. A geometrical model relating the CM to the joint configuration with origin at the toe was expressed through a trigonometric analysis (equation 1):

$$
\begin{aligned}
CM_x(l_i, \theta_i) = {} & m_1 * d_1 * l_1 * cos(\theta_F) + \\
& m_2 * l_1 * cos(\theta_F) + d_2 * l_2 * cos(\theta_F + \theta_A) + \\
& m_3 * l_1 * cos(\theta_F) + l_2 * cos(\theta_F + \theta_A) + d_3 * l_3 * cos(\theta_F + \theta_A + \theta_K) + \\
& m_4 * l_1 * cos(\theta_F) + l_2 * cos(\theta_F + \theta_A) + l_3 * cos(\theta_F + \theta_A + \theta_K) + \\
& \quad d_4 * l_4 * cos(\theta_F + \theta_A + \theta_K + \theta_H) + \\
& m_5 * l_1 * cos(\theta_F) + l_2 * cos(\theta_F + \theta_A) + l_3 * cos(\theta_F + \theta_A + \theta_K) + \\
& \quad l_4 * cos(\theta_F + \theta_A + \theta_K + \theta_H) + d_5 * l_5 * cos(\theta_F + \theta_A + \theta_K + \theta_H + \theta_L) + \\
& m_6 * (l_1 * cos(\theta_F) + l_2 * cos(\theta_F + \theta_A) + l_3 * cos(\theta_F + \theta_A + \theta_K) + \\
& \quad l_4 * cos(\theta_F + \theta_A + \theta_K + \theta_H) + l_5 * cos(\theta_F + \theta_A + \theta_K + \theta_H + \theta_L) + \\
& \quad d_6 * l_6 * cos(\theta_F + \theta_A + \theta_K + \theta_H + \theta_L + \theta_N)
\end{aligned}
\tag{1}
$$

where m_i is the ith segment proportional mass expressed as percentage of total body mass, l_i is the ith segment's length, d_i is the distal distance from the CM of the ith segment expressed as a percentage of its length, where $i = (1, ..., 6) = (foot, shank, thigh, pelvis, trunk, neck)$. The joint angles were primarily used to examine the relation of the elemental variables θ_i with the performance variable CM_x. Displacement of CM_x and joint angle excursion were calculated as the approximate integral of their trajectories.

Components of joint angle variability.

For the present study a variant of the UCM approach, proposed by Scholz et al. [2], was used. Here, the measure of multi-segmental CM control is evaluated at each instant in time to analyze postural responses in different phases during the postural task. For every recorded frame the variance of the control variables (i.e. joint angles) across the attempts can be partitioned into two components: parallel and orthogonal to the UCM (see below). The variance of the performance variable CM orthogonal to the UCM is usually smaller as compared to the variance parallel to it when standing in response to surface perturbation [2]. Both components of joint angle variability were computed to quantify the amount of variability causing unwanted change (task-deviating) and the amount of variability returning the CM to its steady-state position (task-specific). The relative ratio of both components was reported to allow group-wise comparison. Exemplary data is presented in Fig 1.

To obtain the variance of both components, the following steps were applied [2]:

1. Create geometric model (Eq. 1).

2. Compute reference joint-configuration based on mean joint configuration during 1 second prior to perturbation across trials.

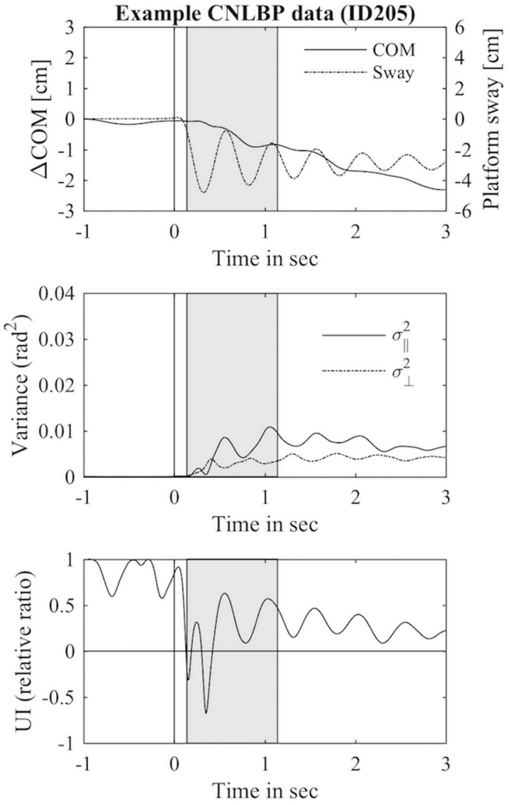

Fig 1. Example data for kinematic analysis. Data of a poorly performing patient ($UI = -.47$). The solid vertical lines indicate the time point of platform release. The gray area represents the active response phase. Top panel shows CM trajectory and actual platform sway trajectory. Middle panel shows normalized variance within and perpendicular to pre-perturbation joint configuration space. Lower panel shows relative ratio of variance.

3. Compute the joint deviation vector (JDV) as the difference between the current joint-configuration and the reference joint-configuration for each segment $\bar{\theta}_i$ at every time-frame of the recording:

$$JDV = \begin{bmatrix} \theta_F - \bar{\theta}_F \\ \theta_A - \bar{\theta}_A \\ \theta_K - \bar{\theta}_K \\ \theta_H - \bar{\theta}_H \\ \theta_L - \bar{\theta}_L \\ \theta_N - \bar{\theta}_N \end{bmatrix} \quad (2)$$

4. Linearise the UCM to relate non-commensurate units with different numbers of degrees of freedom through the definition of the Jacobian matrix $J(\theta)$ and the computation of its null space around the reference configuration, $N(J)$.

$$0 = J(\bar{\theta}) * \epsilon_{n-d} = \begin{bmatrix} \frac{\delta CM_x}{\delta \theta_F} & \frac{\delta CM_x}{\delta \theta_A} & \frac{\delta CM_x}{\delta \theta_K} & \frac{\delta CM_x}{\delta \theta_H} & \frac{\delta CM_x}{\delta \theta_L} & \frac{\delta CM_x}{\delta \theta_N} \end{bmatrix} * \epsilon_{n-d} \quad (3)$$

$$N = \begin{bmatrix} \epsilon_{1F} & \epsilon_{2F} & \epsilon_{3F} & \epsilon_{4F} & \epsilon_{5F} \\ \vdots & \vdots & \vdots & \vdots & \vdots \\ \epsilon_{1N} & \epsilon_{2N} & \epsilon_{3N} & \epsilon_{4N} & \epsilon_{5N} \end{bmatrix} \tag{4}$$

where ϵ_{n-d} are the basis vectors of the null space (n is the number of elemental variables and d is the number of dimensions of the performance variable) representing the linear subspace of all joint-configurations that leave the CM_x position unchanged.

5. Decomposition of the JDV projection into the null-space ($\theta_{||}$ and into its orthogonal space $\theta_{\perp}$:

$$\theta_{||} = \sum_{i=1}^{n-d} \Big(N(J))_i^T \cdot JDV \Big) N(J)_i \tag{5}$$

$$\theta_{\perp} = JDV - \theta_{||} \tag{6}$$

The computed scalar values represent the length of projection to quantify the consistency of the instantaneous joint configuration with the steady-state configuration.

6. Calculate variance normalised to the number of degrees of freedom $(n - d)$ and trial length (N):

$$\sigma_{||}^2 = \frac{\sum_{i=1}^{N} \theta_{||}^2 N}{(n-d)N} \tag{7}$$

$$\sigma_{\perp}^2 = \frac{\sum_{i=1}^{N} \theta_{||}^2 N}{dN} \tag{8}$$

7. Calculate relative variance as UCM-index (UI) with values ranging from -1 to 1 [3]:

$$UI = \frac{\sigma_{||}^2 - \sigma_{\perp}^2}{\sigma_{||}^2 + \sigma_{\perp}^2} \tag{9}$$

References

1. Winter DA. Biomechanics and Motor Control of Human Movement. Hoboken, New Jersey: John Wiley & Sons,; 2009.

2. Scholz JP, Schöner G, Hsu WL, Jeka JJ, Horak F, Martin V. Motor equivalent control of the center of mass in response to support surface perturbations. Experimental brain research. 2007;180(1):163–179.

3. Park E, Reimann H, Schöner G. Coordination of muscle torques stabilizes upright standing posture: an UCM analysis. Experimental brain research. 2016; p. 1–11.
