# Supplementary material for: Postural sensorimotor training versus sham exercise in physiotherapy of patients with chronic non-specific low back pain: An exploratory randomised controlled trial
Source: PLoS One. 2018 Mar 9;13(3):e0193358. doi: 10.1371/journal.pone.0193358 (PMC5844549; doi:10.1371/journal.pone.0193358)
Supplement: S2 Appendix — (PDF) [file pone.0193358.s002.pdf]

 10.1371/journal.pone.0193358

S2 Appendix: Measurement setup

Equipment

Two-dimensional marker trajectories in space were collected at a sampling frequency of 100Hz by two cameras for frontal and sagittal view (1200x720 spatial resolution) [1, 2]. Motion data was recorded with Templo v.8.2 (Contemplas GmbH, Kempten, Germany). Seven sagittal retroreflective markers were applied (mastoid process, shoulder, hip (greater trochanter and anterior superior iliac spine), knee, ankle, and toe. Coordinate data of each reflective marker were filtered at 5 Hz using a bi-directional, second-order, Butterworth digital filter in Matlab™ version R2014b (Mathworks Inc., Natick, MA, USA) [3]. For calibration purposes, fixed geometrical objects with known metrics and fixed angles were placed onto the labile platform and recorded from both perspectives. Centre of pressure (CP) was recorded using the Zebris FDM-S pressure plate (sampling frequency 60 Hz, Zebris Medical GmbH, Isny im Allgaeu, Germany), which was centrally placed on top of the swaying platform (Fig 1). All final analysis algorithms were implemented and executed in Matlab™ version 2014b for Mac (Mathworks Inc., Natick, MA, USA).

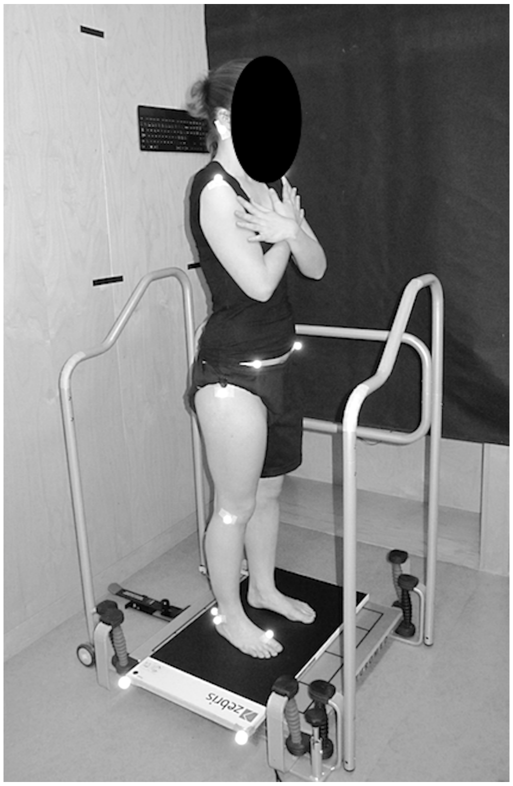

Fig 1. Illustration of the measurement setup (bilateral hip-markers not included in this study).

References

1. Churchill A, Halligan PW, Wade DT. RIVCAM: a simple video-based kinematic analysis for clinical disorders of gait. Computer methods and programs in biomedicine. 2002;69(3):197–209.

2. Fernandes R, Ribeiro J, Figueiredo P, Seifert L, Vilas-Boas J. Kinematics of the Hip and Body Center of Mass in Front Crawl. Journal of Human Kinetics. 2012;33(1):1–9.

3. Scholz JP, Schöner G, Hsu WL, Jeka JJ, Horak F, Martin V. Motor equivalent control of the center of mass in response to support surface perturbations. Experimental brain research. 2007;180(1):163–179.
