# Supplementary material for: Postural sensorimotor training versus sham exercise in physiotherapy of patients with chronic non-specific low back pain: An exploratory randomised controlled trial
Source: PLoS One. 2018 Mar 9;13(3):e0193358. doi: 10.1371/journal.pone.0193358 (PMC5844549; doi:10.1371/journal.pone.0193358)
Supplement: S1 File — (PDF) [file pone.0193358.s003.pdf]

Effects of a sensorimotor training on postural control and pain: A parallel, single-blinded randomised controlled trial

Clinical Study Protocol

Short title: SeMoPoP: Sensorimotor and postural deficiencies in Pain rehabilitation

German title: Effekte eines sensomotorischen Trainings auf posturale Kontrolle und Schmerz: Eine einfachblinde, parallel randomisierte Studie

Study Type:	Clinical trial with Investigational Medical Device (MD)
Study Categorisation:	Risk category A
Study Registration:	www.kofam.ch (SNCTP000000873)<br>www.clinicaltrials.gov (NCT02304120)
Sponsor-Investigator:	Reha Rheinfelden, Research Department,<br>Dr. Corina Schuster-Amft<br>Salinenstrasse 98, 4310 Rheinfelden<br>+41 61 836 53 83<br>c.schuster@reha-rhf.ch
Investigational Product:	Proprioceptive Postural Therapy using the Posturomed, a neuro-orthopaedic therapy device
Protocol Version and Date:	Version 2.0, 31/03/2015

Signature Page

Study number SNCTP000000873

Study Title Effects of a sensorimotor training on postural control and pain: A parallel, single-blinded randomised controlled trial

The Sponsor-Investigator and Principle Investigator have approved the protocol version 2.0 dated 31.03.2015, and confirm hereby to conduct the study according to the protocol, current version of the World Medical Association Declaration of Helsinki, ICH-GCP guidelines or ISO 14155 norm if applicable and the local legally applicable requirements.

Sponsor-Investigator & Principle Investigator: Dr. Corina Schuster-Amft

Rheinfelden, 24.04.2015

Place/Date	Signature

Project leader: Michael A. McCaskey

Rheinfelden, 24.04.2015

Place/Date	Signature

Table of Contents

STUDY SYNOPSIS 7

STUDY SUMMARY IN LOCAL LANGUAGE 10

ABBREVIATIONS 11

STUDY SCHEDULE 12

1. STUDY ADMINISTRATIVE STRUCTURE 13

1.1 Sponsor- and Principle Investigator 13

1.2 Project Leader 13

1.3 Statistician 13

1.4 Laboratory 13

1.5 Monitoring institution 13

1.6 Data Safety Monitoring Committee (DSMC) 13

1.7 Steering Committee (SC) 14

2. ETHICAL AND REGULATORY ASPECTS 15

2.1 Study registration 15

2.2 Categorisation of study (KlinV, Art. 19, 20, App 3, 1.1) 15

2.3 Competent Ethics Committee (CEC) 15

2.4 Competent Authorities (CA) 15

2.5 Ethical Conduct of the Study 15

2.6 Declaration of interest 16

2.7 Patient Information and Informed Consent 16

2.8 Participant privacy and confidentiality 16

2.9 Early termination of the study 17

2.10 Protocol amendments 17

3. BACKGROUND AND RATIONALE 18

3.1 Background and Rationale 18

3.2 Investigational Product (treatment, device) and Indication 19

3.3 Preclinical Evidence 19

3.4 Clinical Evidence to Date 19

3.5 Dose Rationale 19

3.6 Explanation for choice of comparator (or placebo) 20
3.7 Risks/Benefits 20
3.8 Justification of choice of study population 20
4. STUDY OBJECTIVES 21
4.1 Overall Objective 21
4.2 Primary Objective 21
4.3 Secondary Objectives 21
4.4 Safety Objectives 21
5. STUDY OUTCOMES 22
5.1 Primary Outcome 22
5.2 Secondary Outcomes 22
5.3 Other Outcomes of Interest 22
5.4 Safety Outcomes 22
6. STUDY DESIGN 23
6.1 General study design and justification of design 23
6.2 Methods of minimising bias 24
6.2.1 Randomisation 24
6.2.2 Blinding procedures 24
6.2.3 Other methods of minimising bias 24
6.3 Unblinding Procedures (Code break) 24
7. STUDY POPULATION 26
7.1 Eligibility criteria 26
7.2 Recruitment and screening 26
7.3 Assignment to study groups 26
7.4 Criteria for withdrawal / discontinuation of participants 27
8. STUDY INTERVENTION 28
8.1 Identity of Investigational Products (treatment / medical device) 28
8.1.1 Experimental Intervention 28
8.1.2 Control Intervention 28
8.1.3 Packaging, Labelling and Supply (re-supply) 28

8.1.4 Storage Conditions ... 28

8.2 Administration of experimental and control interventions ... 28

8.2.1 Conventional Therapy (both groups) ... 28

8.2.2 Experimental Intervention ... 29

8.2.3 Control Intervention ... 29

8.3 Dose / Device modifications ... 29

8.4 Compliance with study intervention ... 29

8.5 Data Collection and Follow-up for withdrawn participants ... 29

8.6 Trial specific preventive measures ... 29

8.7 Concomitant Interventions (treatments) ... 29

8.8 Study Drug / Medical Device Accountability ... 29

8.9 Return or Destruction of Study Drug / Medical Device ... 29

9. STUDY ASSESSMENTS ... 30

9.1 Experimental procedure ... 31

9.2 Assessments of outcomes ... 31

9.2.1 Assessment of primary outcome ... 31

9.2.2 Assessment of secondary outcomes ... 31

9.2.3 Assessment of other outcomes of interest ... 33

9.2.4 Assessment of safety outcomes ... 33

9.2.5 Assessments in participants who prematurely stop the study ... 34

9.3 Procedures at each visit ... 34

10. SAFETY ... 35

10.1 Drug studies ... 35

10.2 Medical Device Category C studies ... 35

10.3 Medical Device Category A studies ... 35

10.3.1 Definition and Assessment of safety related events ... 35

10.3.2 Reporting of Safety related events ... 35

11. STATISTICAL METHODS ... 36

11.1 Hypothesis ... 36

11.2 Determination of Sample Size ... 36

11.3 Statistical criteria of termination of trial 36
11.4 Planned Analyses....37
11.4.1 Datasets to be analysed, analysis populations....37
11.4.2 Primary Analysis 37
11.4.3 Secondary Analyses 37
11.4.4 Interim analyses....37
11.4.5 Safety analysis....37
11.4.6 Deviation(s) from the original statistical plan 37
11.5 Handling of missing data and drop-outs....37
12. QUALITY ASSURANCE AND CONTROL.... 38
12.1 Data handling and record keeping / archiving....38
12.1.1 Case Report Forms....38
12.1.2 Specification of source documents 38
12.1.3 Record keeping / archiving 38
12.2 Data management....38
12.2.1 Data Management System 38
12.2.2 Data security, access and back-up....39
12.2.3 Analysis and archiving 39
12.2.4 Electronic and central data validation 39
12.3 Monitoring....39
12.4 Audits and Inspections 39
12.5 Confidentiality, Data Protection....39
12.6 Storage of biological material and related health data....39
13. PUBLICATION AND DISSEMINATION POLICY.... 40
14. FUNDING AND SUPPORT.... 41
14.1 Funding 41
14.2 Other Support....41
15. INSURANCE.... 41
16. REFERENCES.... 42

ETH
Eidgenössische Technische Hochschule Zürich
Swiss Federal Institute of Technology Zurich

Reha Rheinfelden

STUDY SYNOPSIS

**Sponsor-Investigator Principle Investigator**	Dr. Corina Schuster-Amft/Ph.D. PT<br>Reha Rheinfelden, Research Department<br>Salinenstrasse 98<br><br>4310 Rheinfelden
**Study Title:**	Effects of a sensorimotor training on postural control and pain: A parallel, single-blinded randomised controlled trial.
**Short Title / Study ID:**	SeMoPoP: Sensorimotor and postural deficiencies in Pain rehabilitation/ 873
**Protocol Version and Date:**	Version 2.0 dated 31/03/2015
**Trial registration:**	www.kofam.ch (SNCTP000000873)<br><br>www.clinicaltrials.gov (NCT02304120)
**Study category and Rationale**	Category A: The medical device is CE marked and will be applied according to the specialised information.
**Clinical Phase:**	Observational study and testing of treatment efficacy of a training method.
**Background and Rationale:**	Sensorimotor training (SMT) is popularly applied as preventive or rehabilitative exercise method in various sports and rehabilitation settings, especially when postural control, linked to proprioception, is suspected to be deficient. Its effect, however, on pain and function is only poorly evaluated.
**Objective(s):**	The projects has two main objectives:<br>1. Measure effects of a four- to five- week sensorimotor training intervention, compared to low intensity endurance training, on musculoskeletal pain, functional status, postural control, and proprioception in patients with chronic non-specific low back pain.<br>2. Evaluate the relationship between cervical proprioception, postural control, and chronic non-specific low back pain (CNLBP) compared to healthy controls.
**Outcome(s):**	**Primary outcomes**:<br>Pain & Functional status: Visual Analogue Scale (VAS) and Oswestry Disability Index (ODI)<br>**Secondary outcomes**:<br>*Postural Control:* Sway of base of support (BOS), centre of pressure of BOS, Centre of Mass, Angular variation of involved segments, Uncontrolled Manifold Index before and after 5-week intervention.<br><br>*Proprioception*: Joint Repositioning Error of cervical, lumbar, hip, knee and ankle joints (before versus after perturbation) and head repositioning sense (HRS) before and after 5-week intervention.

<table>
<tr><td>Study design:</td><td>Randomised, single-blinded, parallel controlled trial evaluation and pre- to post intervention comparison with cross-sectional analysis of baseline parameters for comparison to normative values.</td></tr>
<tr><td>Inclusion / Exclusion criteria:</td><td>Inclusion criteria:
• Patients with clinically confirmed musculoskeletal low back pain (non-specific low back pain)
• VAS pain > 0; ODI>0
• Age ≥ 18 years
Exclusion criteria:
• Known or suspected neurological diseases or lesions
• Traumatic injury of musculoskeletal system (fractures, tumours)
• Spinal pathologies (e.g., tumour, infection, fracture, and inflammatory disease)
• Previous spinal surgery
• Presence of any contraindication to exercise (fracture or cardiovascular limitations)
• Pain in the neck or cervical spine that reduces active movement to less than 30° rotation to each side
• Whiplash during the last year
• Known vestibular pathologies
Healthy group:

Participants of the healthy group must be free of neurological or orthopaedic limitations to daily activity (VAS=0; ODI=0).</td></tr>
<tr><td>Measurements and procedures:</td><td>Interested individuals will be informed (written and personally) and given at least 24 hours of consideration time before being invited to baseline (BL) measurements.

Healthy participants will be invited for a single baseline measurement of all primary and secondary outcomes.

Patients with CNLBP will be invited to baseline measurements after which they may still decide whether or not they would like to take part in the RCT trial. After a second assessment (pre-intervention, T_0), included patients will then be randomly allocated to either the experimental (EG) or the control group (CG). All participating patients will receive standard physiotherapy. CG will receive additional exercise therapy (LIT), the EG will receive additional SMT.

One or two days after (T_1) and four weeks after (FU) the last treatment, the project leader (PL) or the technical assistant will record the post-intervention assessment of the primary and secondary outcomes.

Each assessment will consist of a postural control task (standing on a labile platform and stabilising anticipated anterior-posterior sway), a head-repositioning task, and two questionnaires. Total duration will be approximately one hour.</td></tr>
<tr><td>Study Product / Intervention:</td><td>SMT will be performed with the Posturomed®. The Posturomed is a neuro-orthopaedic therapy device used in rehabilitative and exercise settings to prevent muscular imbalance and promote restitution of functional stability. A 7-stage protocol exists and is instructed by exercise therapists. This particular form of SMT is henceforth termed Postural Proprioceptive Therapy (PPT). Recommendations for home exercise will be provided.</td></tr>
</table>

**Control Intervention (if applicable):**	PPT will be compared to a sham intervention (low-intensity endurance training).
**Number of Participants with Rationale:**	20 to 30 patients with low back pain (min. 10 per arm) and 20 healthy controls for the normative data. Based on previous studies with similar endpoints, the selected amount of participants has shown to suffice for an explorative approach with a desired power of 80% and an alpha of 0.05.
**Study Duration:**	September 2014 to December 2015
**Study Schedule:**	Begin recruiting: January 2015<br>Last assessment: December 2015
**Investigator and project leader:**	Michael A. McCaskey<br>m.mccaskey@reha-rhf.ch<br>+41 61 836 53 83
**Study Centre:**	Reha Rheinfelden<br>Salinenstrasse 98<br>4310 Rheinfelden
**Statistical Considerations:**	Student T-Test will be applied for group comparison at Baseline for cross-sectional comparison of LBP group with healthy group.<br>Repeated measure ANOVA with post-hoc analysis will be applied to test therapy effects.<br>Effect size will be calculated with Guyatt's effect size.
**GCP Statement:**	This study will be conducted in compliance with the protocol, the current version of the Declaration of Helsinki, the ICH-GCP or ISO EN 14155 (as far as applicable) as well as all national legal and regulatory requirements.

STUDY SUMMARY IN LOCAL LANGUAGE

Effekte eines sensomotorischen Trainings auf posturale Kontrolle und Schmerz: Eine einfachblinde, parallel randomisierte Studie

Chronischer Rückenschmerz ist nach wie vor die häufigste Ursache verminderter Lebensqualität und steigenden Arbeitsausfällen, was mit hohen sozioökonomischen Kosten verbunden ist. Sensomotorisches Training wird dabei sehr häufig in der Schmerzrehabilitation eingesetzt, da angenommen wird, dass das Schmerzempfinden mit der Körperwahrnehmung und Haltungskontrolle zusammenhängen. Eine Verbesserung der Körperwahrnehmung durch Sensomotorisches Training soll so zur Reduktion der Schmerzen und auch zur verbesserten Haltungskontrolle führen. Bisher wurden aber noch keine Studien durchgeführt, welche die Effektivität dieser Trainingsmethode untersuchten.

Diese Studie verfolgt zwei Ziele. Einerseits sollen die Haltungskontrolle und die Körperwahrnehmung zwischen Gesunden und Patienten mit chronischen Rückenschmerzen verglichen werden. Zur Messung der Haltungskontrolle wird ein Parameter vorgestellt, der es ermöglichen soll, die zeitabhängige Veränderung der Haltung zu erfassen. Bisherige Messmethoden erlauben nur eine statische Analyse der Haltung, die wenig Aussagen zur funktionellen Haltungskontrolle erlauben. Um die Körperwahrnehmung zu vergleichen, wird die Fähigkeit untersucht, eine vorgegebene Kopfposition mit geschlossenen Augen einzunehmen.

Als zweites Ziel soll die Wirksamkeit einer sensomotorischen Trainingsmethode auf labiler Unterlage untersucht werden (posturale propriozeptive Therapie). Patienten, die nach der beschriebenen Erstuntersuchung an der Interventionsstudie teilnehmen möchten, werden zufällig in eine von zwei Trainingsgruppen eingeteilt. Beide Gruppen erhalten neun Einheiten konventioneller Physiotherapie die jeweils 30 Minuten dauern. In der Experimentalgruppe wird zusätzlich ein 15-minütiges sensomotorisches Training angeboten. Die Kontrollgruppe erhält niederschwelliges Ausdauertraining derselben Dauer. Um allfällige Veränderungen zu messen, werden Parameter der Haltungskontrolle und das persönliche Schmerzempfinden sowie Einschränkungen im Alltag der Patienten erfasst. Für die Studie werden insgesamt vier Messungen stattfinden: Erstuntersuchung beim Vergleich mit gesunden, Zweituntersuchung zum Ermitteln der Ausgangswerte unmittelbar vor der ersten Therapie, Nachfolgeuntersuchung unmittelbar nach der letzten Therapie sowie eine weitere Nachkontrolle vier Wochen nach der Intervention.

ABBREVIATIONS

Provide a list of abbreviations used on the protocol - to be completed

AE	Adverse Event
BL	Baseline
BOS	Base of support
CEC	Competent Ethics Committee
CG	Control group
CNLBP	Chronic non-specific low back pain
COM	Centre of mass
COP	Centre of pressure
CRF	Case Report Form
DSMC	Data Safety Monitoring Commettee
EG	Experimental group
EKNZ	Ethikkommission Nordwest-und Zentralschweiz
GCP	Good Clinical Practice
H0	Null hypothesis
H1	Alternative hypothesis
HFG	Humanforschungsgesetz (Law on human research)
IIT	Investigator-initiated Trial
IMP	Investigational Medicinal Product
ISO	International Organisation for Standardisation
ITT	Intention to treat
LBP	Low back pain
LIT	Low-intensity endurance training
MD	Medical Device
ODI	Oswestry Disability Index
PL	Principal Investigator
PL	Project Leader
PPT	Postural proprioceptive training
SDV	Source Data Verification
SMT	Sensorimotor training
SOP	Standard Operating Procedure
SPC	Summary of product characteristics
SUSAR	Suspected Unexpected Serious Adverse Reaction
T0	Assessment before intervention
T1	Assessment directly after intervention
T2	Assessment 4 weeks after end of intervention
TMF	Trial Master File
UCM	Uncontrolled Manifold
VAS	Visual Analogue Scale

STUDY SCHEDULE

Table 1: Study procedure

Study events	*Recruiting*	*Baseline(BL)*	*Pre-intervention (T0)*	*Intervention*	*Post-intervention (T1)*	*Follow-up (T2)*
Visit<br>Duration	0 (phone)<br>1x20‘	1<br>1x60‘	2<br>1x60‘	3-12<br>9x45‘	13<br>1x60‘	14<br>1x60’
Time	1st week	1st week	1st week	1st to 5th week	5th week	9th week
Patient information	x					
Written consent		x				
Socio-demographic characteristics		x				
Medical history	x					
Eligibility	x	x				
Randomisation				x		
Therapies				x		
Primary outcomes		x	x		x	x
Secondary outcomes		x	x		x	x
Documentation of adverse events			x	x	x	x

1. STUDY ADMINISTRATIVE STRUCTURE

1.1 Sponsor- and Principle Investigator

Institution:	Reha Rheinfelden, Clinical Research Department
Contact:	Dr. Corina Schuster-Amft
Title and Position:	Ph.D., MPTSc., Head of Research Department
Contact details:	Reha Rheinfelden, Salinenstrasse 98, 4310 Rheinfelden<br>+41 61 8365383<br>c.schuster@reha-rhf.ch

1.2 Project Leader

Name:	Michael A. McCaskey
Title and Position:	M.Sc. ETH Human Movement Science, Research Associate and Ph.D. candidate
Contact details:	Reha Rheinfelden, Salinenstrasse 98, 4310 Rheinfelden<br>+41 61 8365381<br>m.mccaskey@reha-rhf.ch

1.3 Statistician

The PL will conduct the final analysis. However, the PL will make use of the statistical services from the ETH Zurich and will uphold regular contact with the clinic's statistical advisor, Dr. M. Stöckli (University of Basel).

**ETH Zurich**	Statistical Consulting Service, Seminar for Statistics, HG G 10.3<br>Rämistrasse 101, 8092 Zurich, Switzerland<br>+41 44 632 2223<br>beratung@stat.math.ethz.ch
**Reha Rheinfelden**	Dr. Markus Stöckli, University of Basel, Institute for Psychology<br>Missionstr. 62A, 4055 Basel<br>+41 61 267 3530<br>markus.stoecklin@unibas.ch

1.4 Laboratory

Not applicable.

1.5 Monitoring institution

Please refer to section 1.1.

1.6 Data Safety Monitoring Committee (DSMC)

As the trials involves only minimal risks, is conducted with marketed and CE certified medical products, and is of only short duration, it is deemed not necessary to establish a DSMC.

1.7 Steering Committee (SC)

Dr. Corina Schuster-Amft
Reha Rheinfelden, Reha Rheinfelden
+41 61 836 5383; c.schuster@reha-rhf.ch

Prof. Dr. Nicole Wenderoth
ETH Zurich; Neural Control of Movement Lab, D-HEST
+41 44 635 6157; nicole.wenderoth@hest.ethz.ch

PD Dr. Eling Douwe de Bruin
ETH Zurich; Institute for Human Movement Science, D-HEST
+41 44 632 4018; eling.debruin@hest.ethz.ch

2. ETHICAL AND REGULATORY ASPECTS

The decision of the CEC and Swissmedic/foreign competent authority concerning the conduct of the study will be made in writing to the Sponsor-Investigator before commencement of this study. The clinical study can only begin once approval from all required authorities has been received. Any additional requirements imposed by the authorities shall be implemented.The clinical study will only begin after written approval of the CEC has been received.

2.1 Study registration

The study protocol has been pre-registered on the Swiss Federal Complementary Database (www.kofam.ch, SNCTP000000873) and will be completed when CEC approval is given. Additionally, the trial has been registered at www.clinicaltrials.gov (NCT02304120) and the study protocol will be submitted for publication in suitable peer-reviewed journals (e.g. BMC trials).

2.2 Categorisation of study (KlinV, Art. 19, 20, App 3, 1.1)

The clinical trial and observational study corresponds to the Category A, as the product is authorised in Switzerland and the application is done according to the specific information.

2.3 Competent Ethics Committee (CEC)

The responsible investigator (PL) will ensure that approval from CEC (EKNZ) is sought for the clinical study.

The PL is also responsible to uphold a weekly progress report that tracks number of:

- Screened patients
- Contacted participants
- Invited participants
- Recruited participants
- Drop-outs
- Measurement events
- Changes in research activity (setup, amendments)
- Unanticipated problems involving risks to humans
- Planned or premature study end

Also, a final study report will be submitted to the CEC after completion of the investigations. No changes will be made to the protocol without prior Sponsor and CEC approval, except where necessary to eliminate apparent immediate hazards to study participants.

Premature study end or interruption of the study will be reported within 15 days. The regular end of the study will be reported to the CEC within 90 days, the final study report shall be submitted within one year after study end. Amendments are reported according to chapter 2.10.

2.4 Competent Authorities (CA)

Not applicable. There are no other authorities involved in this study.

2.5 Ethical Conduct of the Study

The study will be carried out in accordance to the protocol and with principles enunciated in the current version of the Declaration of Helsinki, the guidelines of Good Clinical Practice (GCP) issued by ICH and the European Directive on medical devices 93/42/EEC and the ISO Norm 14155 and ISO 14971, the Swiss Law and Swiss regulatory authority’s requirements. The CEC and regulatory authorities will receive annual safety and interim reports and be informed about study stop/end in agreement with local requirements.

2.6 Declaration of interest

The PL is employed by the participating clinic (Reha Rheinfelden). The project is also financially supported by the ETH Zurich (Institute for Human Movement Science) for material and travel expenses. The steering committee declares there is no conflict of interest, whether intellectually or financially. There is no financial dependence from the developer of the therapeutic device; the company is not involved in the study initiation, planning, conducting, or data analysis and interpretation.

2.7 Patient Information and Informed Consent

The investigators will explain to each participant the nature of the study, its purpose, the procedures involved, the expected duration, the potential risks and benefits and any discomfort it may entail. Each participant will be informed that the participation in the study is voluntary and that he/she may withdraw from the study at any time and that withdrawal of consent will not affect his/her subsequent medical assistance and treatment.

The participants of the intervention trial will also be informed that allocation to either treatment group will be based on randomisation and cannot be influenced. He or she must also know that if the baseline measurements are contraindicative to the treatment, exclusion of the intervention study may be possible after the second measurement event (T_0), i.e. prior to begin of the intervention.

The participant will be informed that his/her medical records may be examined by authorised individuals other than their treating therapist.

All participants for the study will be provided a participant information sheet and a consent form describing the study and providing sufficient information for participant to make an informed decision about their participation in the study. The participants will have at least 24 hours to decide whether to participate or not.

The patient information sheet and the consent form will be submitted to the CEC and to the competent authority (as applicable) to be reviewed and approved. The formal consent of a participant, using the approved consent form, must be obtained before the participant is referred to any study procedure.

The participant will read and consider the statement before signing and dating the informed consent form, and will be given a copy of the signed document. The consent form will also be signed and dated by the investigator and it will be retained as part of the study records.

The participants of the study will not be compensated financially for their participation, but expenses will be reimbursed with CHF 10.00 per visit. Additionally, three entries to a local Spa-centre and one wellness massage will be raffled among all participants.

2.8 Participant privacy and confidentiality

The investigator affirms and upholds the principle of the participant's right to privacy and that they shall comply with applicable privacy laws. Especially, anonymity of the participants shall be guaranteed when presenting the data at scientific meetings or article publications in scientific journals.

Individual subject medical information obtained as a result of this study is considered confidential and disclosure to third parties is prohibited. Subject confidentiality will be further ensured by utilising subject identification code numbers to correspond to treatment data in the computer files.

For data verification purposes, authorised by representatives of the Sponsor-Investigator, members of the ethics committee may require direct access to parts of the medical records relevant to the study, including participants' medical history.

2.9 Early termination of the study

The Sponsor-Investigator may terminate the study prematurely according to certain circumstances, for example:

- ethical concerns,
- insufficient participant recruitment,
- when the safety of the participants is doubtful or at risk, respectively,
- alterations in accepted clinical practice that make the continuation of a clinical trial unwise,
- early evidence of benefit or harm of the experimental intervention.

2.10 Protocol amendments

Only the PL can make amendments to the protocol but may designate technical assistants or study nurses to report changes to the CEC. The steering committee will be in continuous contact with the PL to suggest changes to the procedures, if necessary (e.g. eligibility criteria).

Substantial amendments are only implemented after approval of the CEC.

Under emergency circumstances, deviations from the protocol to protect the rights, safety and well-being of human subjects may proceed without prior approval of the sponsor and the CEC. Such deviations shall be documented and reported to the sponsor and the CEC as soon as possible.

All Non-substantial amendments are communicated to the CEC within the Annual Safety Report (ASR).

3. BACKGROUND AND RATIONALE

3.1 Background and Rationale

In 2006, the European Cooperation in Science and Technology working group B13 (COST B13) published guidelines for chronic low back pain (CLBP) treatment [1]. The report estimates the lifetime prevalence to experience a first ever episode of low-back pain (LBP) at 84%. The State Secretariat for Economic Affairs (SECO) in Switzerland has released corresponding numbers in the context of preventive measures for occupational settings. According to the author, 18 % of all employees in Switzerland have reported some form of work related back pain accounting for 26 % of occupational absence with corresponding socio-economic consequences [2]. Although the bulk of the direct costs have been attributed to care by medical physicians and non-physicians [3], it is the indirect costs through absenteeism and social isolation that cause more than 80% of health costs. Hence, research promoting return to normal activity and prevention of chronicity of pain remains of great importance.

Where pain persists for more than 12 weeks and cannot be attributed to a recognisable, known specific pathology, the diagnosis is often chronic non-specific low back (CNLBP, ICD 54.5). This is the case in 80% of all pain patients referred to physiotherapy [1, 4]. Consequently there has been extensive research activity on the topic of CNLBP significantly contributing to the understanding of pain [5].

A proposed model describes the lack of variability as a possible cause for posture related stress to the musculoskeletal system [6-9]. In modern society accurate responses to dynamic movements are becoming ever more neglected. Repetitive tasks seem to dominate most of our activities. It has been well established that occupations requiring prolonged periods of static standing are associated with development of musculoskeletal disorders including LBP [10-12]. Long-term monotonous afferent input is believed to impair the sensorimotor system. Circuits regulating the appropriate amount of symmetric muscle force, needed to adapt the correct posture in any given situations, are thought to be disturbed [2, 8, 9, 13, 14]. If not restored, this constant malfunctioning of muscular control and regulation of dynamic movement may lead to inappropriate muscular activity [2, 14, 15] and is thought to contribute to taut muscles, imbalanced muscle activation, poor posture, and ultimately to musculoskeletal pain in lumbar regions.

Consequently, neuromuscular rehabilitation techniques addressing sensory deficiencies have emerged in recent years and have received increasing therapeutic attention [8, 9, 15-17]. These techniques could broadly be summarised as sensorimotor methods aiming at increased proprioceptive input to improve motor response to changing environments [15, 16]. It is believed that sensorimotor training (SMT) increases afferent proprioceptive stimulation of the segmental coordination through coupled anticipatory feed forward tasks and supervised postural response. This might lead to improved quality of postural control, which in turn may ultimately alleviate postural specific musculoskeletal pain.

There has been some doubt on whether such SMT can actually improve proprioceptive acuity in a functional way at all. In a recent review, Ashton-Miller et al. outlined a row of concerns (e.g. lack of neurophysiological evidence) about the validity of current proprioceptive exercises [18]. Although many therapists and clinicians report successful treatment cases, the exact effect and validity of sensorimotor interventions is still discussed controversially [18, 19]. Thus, the European Guidelines on the management of CNLBP do not include recommendations for SMT [1].

To assess the validity of these claims there is a need for outcomes that are capable of measuring postural motor control outcomes reliably and accurately in relation to pain and proprioception. Questionnaires for pain assessment are limited to self-reported indication of pain level which is a subjective measure. Functional assessment of postural control is also limited to clinical observation or outcomes that may be related to but not caused by motor control. Quantitative assessments e.g. COP, may be an objective and reliable tool for postural control assessment, but fails to describe the segmental coordination and ignores the individual variability inherent to human movement. Human posture requires complex mechanisms to perform the simplest of movements. Changing only one of the incoming control parameters leads to adaptation on all segments of the body, albeit not always detectable. This complexity is not captured with linear methods (e.g. mean and standard deviation of COP) [20, 21]. As Harbourne et al. point out, redundancy cannot be neglected when assessing the

quality of a task, as it is redundancy that allows the countless options humans have to react to varying perturbations [21].

The study has two interrelated aims. First, an experimental measurement setup is proposed that allows quantitative nonlinear analysis of COP and joint configuration during a dynamic postural task. The relationship of the proposed outcome with cervical proprioception is analysed and compared to healthy controls. Second, the efficacy of added proprioceptive training in conventional physiotherapy is assessed using the proposed parameters as secondary outcomes.

3.2 Investigational Product (treatment, device) and Indication

There is a wide variety of ways how SMT can be performed. For this study, proprioceptive postural training (PPT) will be applied using the neuro-orthopeadic therapy device Posturomed®. The Posturomed consists of a labile platform, with adjustable damped swaying behaviour. Media-lateral and anterio-posterior sway are increased when the two brakes, one at the front and one at the back, are released. This gives it three specific configurations with increasing levels of instability. The Posturomed® is used for therapy, but has also been used for assessment of postural [22]. In contrast to most proprioceptive training devices, the exercise plan for PPT is clearly defined, quickly explained to the patient and easily understood.

PPT is indicated for postural specific back pain, functional instability of weight-bearing joints (e.g. knee- or ankle instability), hypermobility, and other postural deficiencies. PPT is never applied as a stand-alone therapy but always embedded in multimodal therapy.

3.3 Preclinical Evidence

Not applicable (marketed device).

3.4 Clinical Evidence to Date

PPT will be performed using the neuro-orthopaedic therapeutic device Posturomed®. It was marketed in 1993 by a developer of orthopaedic chairs, Haider Bioswing, in collaboration with specialists from physical rehabilitation of pain [9]. It has been used as postural measure balance in a row of intervention studies after its physical characterisation and discriminant validity was confirmed [22, 23]. Combined with a non-contact path measuring system the therapeutic device is deemed to be suitable for characterizing balance abilities in an upright one-legged stance. Low variability of oscillation frequency suggests mechanically caused sway whereas the extent and duration of the oscillation is believed to be caused by the neuromuscular regulation of the subject. Hence, the Posturomed® can be combined with an accelerometer to quantify balance [22, 23].

In an unpublished thesis by Ganz et al. [24], postural control was significantly improved and reduced pain perception and functional impairment were reported. However, co-interventions were not controlled and group allocation was not randomised. Together with the small sample size applied, it is not possible to make any conclusive remarks on the clinical evidence for sensorimotor Training (SMT) effectiveness. Also, after conducting a systematic review on the topic of general SMT in pain rehabilitation [25], it was shown that no high-quality studies exist and that there is inconsistent evidence on the benefits of SMT.

3.5 Dose Rationale

In a systematic review by McCaskey et al. [25] it was shown that no recommendations for dose selection for SMT implementation are available. Dose, frequency, and duration of SMT are still very much disputed, as sensory adaptation cannot be separated from motor learning. Dose selection for the current study is based on instructions of the usage of the medical device and on training principles in general.

3.6 Explanation for choice of comparator (or placebo)

Low intensity endurance training (LIT) does not provide a specific treatment effect to the sensorimotor system but can improve the global perception of well-being. Although no cardiovascular response is expected at these low intensities, LIT can help to gradually increase the level of activity and participation and is therefore recommended as part of CNLBP treatments [4]. Moreover, LIT is easily standardised and monitored throughout the trial duration.

3.7 Risks/Benefits

The exercises on the labile platform are rated as "absolutely safe" by the developer. Ever since the therapy has been implemented (1993), no adverse events have been reported during or after training on the device. The labile surface may trigger some fear of falling, but the railings that surround the device allow control of stability at any time. Nevertheless, it is not recommended to use the device during acute phases of intense pain or inflammation of weight bearing joints (e.g. knee or hip joints). Also, severe vestibular deficits or M. Menière may also lead to inability to stand stable and securely on the platform. To mitigate these risks, eligibility criteria will exclude participants with any risk-bearing contra-indications.

Although not confirmed systematically, the benefits expected outweigh the minimal risks by far. It is expected that an overall improvement of balance and postural control improves reactivity and reduces risk of falling in daily activity. Specific improvement of inter-vertebral musculature and improvement of the fine-tuned control of posture is believed to relax tensed poly-segmental muscles and decrease long-term strains and pain.

Patients that are enrolled into the study are covered by indemnity for non-negligent harm associated with the protocol through the clinic's own insurance (National Suisse, police-nr: 40.025.077). This will cover compensation or damages whether awarded voluntarily by the Sponsor, or by claims pursued through the courts. Incidences judged to arise from negligence (including those due to major protocol violations) will not be covered by study insurance policies.

If patients feel they benefited from the additional PPT therapy, additional training sessions can be organised for the patient. However, this will be outside of the study and will require either a new referral from the treating physician or the costs must be beard by the patient him- or herself.

Irrespective of treatment success, the study shall produce new insight into the understanding of the mechanisms behind human postural control and its relation to musculoskeletal pain.

3.8 Justification of choice of study population

To study the effects of proprioceptive training, a population of chronic non-specific low back pain patients will be invited. As described above (Background and Rationale), the sensorimotor control is believed to be reduced in chronic low back pain. Hence it makes sense to test motor control and the efficacy of a sensorimotor training program with the suggested study population.

4. STUDY OBJECTIVES

The overall objective of the study is to evaluate the effectiveness of a widely used but poorly investigated treatment method for non-specific low back pain rehabilitation. Secondary aims are to propose a measurement setup for dynamic postural control assessment and to provide insight into associations of postural deficiencies with proprioceptive accuracy and pain.

The three main research questions are:

1. Does a 5-week sensorimotor training intervention improve musculoskeletal pain and postural control?
2. Do patients with CNLBP present with poorer postural control and cervical proprioception than healthy controls?
3. Is there a relationship between cervical proprioception and postural control?

4.1 Overall Objective

The purpose of this study is to evaluate the relationship between low back pain, postural control and cervical proprioception. Furthermore it will be shown whether additional sensorimotor training can effectively improve rehabilitation outcomes in patients with CNLBP.

4.2 Primary Objective

The study seeks primarily to determine the effect of SMT on pain and functional status when added to conventional physiotherapy compared to usual treatment of patients with CNLBP.

4.3 Secondary Objectives

Secondary objectives are to test dynamic postural control and cervical proprioception in a clinical setting with a simple motor task. The motor task performance of patients with CNLBP will be compared with healthy controls. The motor task for postural control will be to recover normal standing on a labile platform after a slight platform perturbation. The setup measures dynamic control of posture. For cervical proprioception, head repositioning acuity will be measured.

4.4 Safety Objectives

The study’s safety objectives are to systematically record any adverse events in order to assess the safety of SMT in the clinical setting.

5. STUDY OUTCOMES

5.1 Primary Outcome

The primary endpoint will be the functional status to assess impairment of daily activities and the self-reported pain after the intervention.

5.2 Secondary Outcomes

Secondary endpoints will be the assessment of the quality of postural control (centre of pressure, platform sway and Uncontrolled Manifold Index (UCM)), and the proprioceptive acuity of cervical repositioning sense (head-repositioning error).

5.3 Other Outcomes of Interest

Therapy documentations will also be analysed qualitatively to compare the amount of active and passive treatments applied during the conventional physiotherapy sessions. Particularly the physiotherapist's patient diagnosis will be extracted to compare with the outcome of postural control. Congruency of prediction could be analysed, i.e. does the dynamic analysis of postural control suggest similar postural deficiencies as described by the therapist?

5.4 Safety Outcomes

A weekly reporting scheme will document progress of the study. Any occurrences will be recorded to the sheet, including adverse events or identified potential risks.

6. STUDY DESIGN

6.1 General study design and justification of design

The intended procedures and study stages are summarised in Table 1 and Figure 1. Patients will be recruited from the out-patient department of the study site (Reha Rheinfelden) and public advertisement. In a cross-sectional study, baseline (BL) values are recorded to assess and to identify expected discrepancies in postural behaviour in a group-wise comparison (healthy vs. patients with LBP). In a pilot randomised sham-controlled trial (RCT) with parallel groups and blinded assessors, the effects of added SMT to conventional therapy in comparison to added LIT will be evaluated on three additional measurement events. The primary endpoints are pain and function before (T_0) and after 9 treatments during 5 weeks (twice a week). Short-term effects will be evaluated within a week after the last training session (T_1). Long-term effects will be tested four weeks after the last training session (T_2).

Screening and pre-intervention assessments: Potential participants will be informed (personal or by phone and email) of the study procedures, risks and expenses, and possible benefits (according to the patient information sheet approved by the competent ethical committee, CEC). After written and oral information, the patient will have one to three days to consider participation before giving informed consent. Once written consent is provided, a baseline measurement will take place 2 to 4 days prior to therapy commencement. For healthy participants there is no time constraint. An internal rheumatologist will confirm the diagnosis for patients who are not referred to the clinic by their treating physicians. The BL assessment will reveal whether all eligibility criteria are fulfilled and whether trial participation is granted. Patients will also be asked to give a second oral consent to further participate in the trial. Once included and randomised for treatment groups, all analysis will follow intention-to-treat principles (as-per-case analysis will also be conducted). T_0 will be conducted immediately before first treatment to ensure a stable baseline (4 to 7 days after BL).

Interventions and follow-up assessments: Patient allocation to either the sham- or the experimental PPT group will be block-randomised with a 1:1 allocation ratio. The sequence list will be guarded by the central pharmacy (central randomisation list). Before the first treatment begins, the treating therapist will call the pharmacy to learn the allocated group belonging to the patient's identification number. All patients will receive usual physiotherapy as deemed appropriate by the therapist for 30 minutes. Patients allocated to the CG will subsequently be instructed on how to use the treadmill, stationary bike, or cross-fit trainer at low intensity (Borg 6-9). Patients in EG will receive supervised instructions to use the therapy device (Posturomed®) correctly. For treatments 2 to 9, patients in both groups will not be supervised directly but a trained therapist will be nearby to oversee the compliance and correct execution of the exercise. The therapist will evaluate quality of the execution of exercises after the 3rd and 6th session to instruct increased difficulty if the patient already masters the task. Within one or two days after the 9th treatment, the second assessment (T_1) of primary and secondary outcomes will be conducted by the PL or a technical assistant to record possible short-term effects. Finally, four weeks after the last treatment a follow-up measurement will assess long-term effects of the treatments. All measurement events (ME) are expected to take about 60 minutes for all outcome assessments. Total therapy duration is 45 minutes (30 minutes therapy and 15 minutes experimental or sham intervention). The therapist will be blinded to the results of all primary and secondary outcomes. The assessor will remain blinded to group-allocation. For description of the sequence generation and randomisation procedures, please refer to section 6.2.

Limitations: The study-protocol features some technical, methodological and physiological limitations that must be considered when interpreting the results. First, the physiological limitation concerns the indirect measure of proprioception which limits the interpretation regarding increased afferent input. Joint repositioning sense only tests one aspect of proprioception on a conscious level. Second, from a methodological point of view, it is not possible to blind the therapist or the patient to the intervention. This may influence the way patients respond to subjective outcomes and could potentially bias the results. Furthermore, in a clinical setting it is not possible to control all co-interventions as treatment must be adapted to the needs and indications of each patient. With multiple therapists at work standardisation of the experimental intervention is adamant. Third, the recording of non-active markers is always related to some inaccuracies. The measurement device records with a resolution that allows accuracy of about 3 mm. This may limit the detectability of the expected change in postural control.

A further methodological limitation is that both assessment for secondary outcomes and the experimental intervention involve the same device (Posturomed). However, the task for the assessment deviates substantially from the training task and no advantage is expected for EG group when performing the postural control task (described in section 9.2.2).

6.2 Methods of minimising bias

6.2.1 Randomisation

Participants will be randomly assigned to either control or experimental group with a 1:1 allocation as per the computer generated randomisation schedule using permuted blocks of random sizes. The block sizes will not be disclosed, to ensure concealment. A technical assistant otherwise not involved in the study will create the randomisation list, which will be stored in the clinic's pharmacy.

6.2.2 Blinding procedures

The assessor will remain uniformed about group allocation. During the assessment, the participating patients will repeatedly be reminded not to mention treatment group during assessments. The list with sequential participant numbers and randomised allocation will be stored out of sight of the assessor. Concealment will remain after study finalisation for data analysis. Both treatments, control and experimental, will take place in the same room as a measure against the likelihood that the PL would be able to guess group allocation when accidentally seeing the participant in the corridors.

6.2.3 Other methods of minimising bias

The questionnaires for pain and functional status are all standardised and self-ministered.

Prior to study commencement, the involved therapists will attend a standardisation training where the exact execution and documentation of the intervention is trained. This will also entail standardisation of conventional treatment to the extent of possibilities within therapeutic practicability.

6.3 Unblinding Procedures (Code break)

The PL and assessors will never be unblinded to the patient allocation as they are otherwise not involved treatment of the patients. As therapists and medical staff are not blinded from the first place, there is no protocol for unblinding procedures.

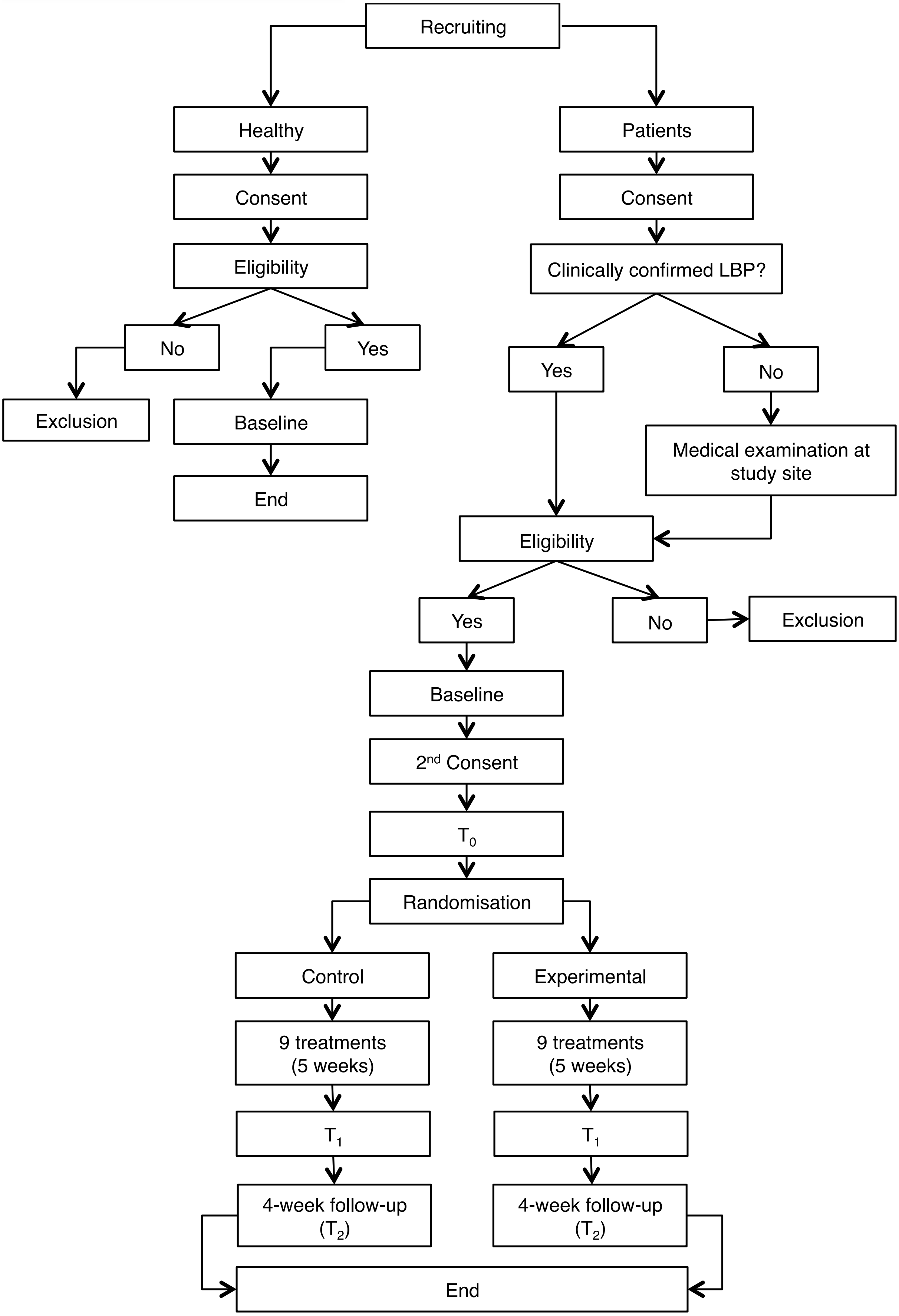

Figure 1: Study procedure flow-chart

7. STUDY POPULATION

7.1 Eligibility criteria

Participants fulfilling all of the following inclusion criteria are eligible for the study:

- Patients with musculoskeletal low back pain (lumbosacral pain, with or without radicular pain).
- Pain and discomfort localized below the costal margin and above the inferior gluteal folds, with or without referred leg pain of at least 3 months' duration.
- Currently seeking care for low back pain.
- Age above 18 years.
- Comprehend German.
- Informed Consent as documented by signature (Appendix Informed Consent Form).

The presence of any one of the following exclusion criteria will lead to exclusion of the participant:

- Clinical signs of neurological damage (i.e. paresis or tingling in limbs).
- Traumatic injury to the musculoskeletal system, such as fractures or tumours.
- Suspected or confirmed spinal pathology (e.g., tumour, infection, fracture, and inflammatory disease).
- Previous spinal surgery.
- Major surgery scheduled during treatment or follow.
- Pain in the neck or cervical spine that reduces active movement to less than 30° rotation to each side
- Whiplash during the last year
- Known vestibular pathologies
- Inability to follow the procedures of the study, e.g. due to language problems, psychological disorders, dementia, etc. of the participant.
- Parallel participation in another study.
- Previous enrolment into the current study.
- Enrolment of the investigator, his/her family members, employees and other dependent persons.

7.2 Recruitment and screening

The therapy management office will notify the PL when patients matching the inclusion criteria are referred to the outpatient department of the study site. The time lapse between referral and first treatment appointment is approximately 2 weeks. During this time the PL will contact the patient to inform about the study and invite him or her for a baseline assessment. Additionally, local care providers and physicians will be contacted and information leaflets provided to the public (online media, print media, local health institutes and Spa-Centres). These flyers will not be distributed before ethical approval for their content is given. Interested participants will contact the PL to receive detailed information (as described above). If the patient was not referred to study site by an external physician, they must first be examined by the clinic's internal rheumatologist to confirm diagnosis of chronic lumbosacral spinal pain (including radicular pain).

Healthy participants will be invited through homepage advertisement, local media, and internal pin-board (for staff members).

7.3 Assignment to study groups

A technical assistant, otherwise not involved in the study, will compute the blocked randomisation sequence. The sequentially numbered list will be kept out of reach from the PL. Allocation remains concealed to the outcome assessor (see Figure 1 and Table 1).

7.4 Criteria for withdrawal / discontinuation of participants

The measurement event or intervention will be immediately terminated if any one of the following events occurs:

- Participant refuses to give or withdraws written consent.
- Participant decides to end measurements or intervention for any given reason.
- Project leader or therapist notices uneasiness of the participant during assessments or during experimental intervention (e.g. dizziness or unexpected increase of pain level).
- Sustained increase of symptoms after training sessions after the first 9 sessions.
- Participant is injured after fall or other trauma (e.g. fracture).

8. STUDY INTERVENTION

8.1 Identity of Investigational Products (treatment / medical device)

8.1.1 Experimental Intervention

The experimental group will receive added PPT to the conventional physiotherapy. PPT will be applied by means of a neuro-orthopaedic medical device (Posturomed®, see section 3.2). The Posturomed allows adaptive oscillation in the horizontal plane. Therapy instructions advise seven stages of difficulty. In all stages the patient is asked to provoke oscillation by stepping on site. After three steps, the patient must stand still on one leg for 2 seconds before he or she repeats the steps. Difficulty is increased by a) decreasing the damping through release of the breaks and b) through added juggling of a ball during the motor task (dual-task and divided attention). The next stage is reached once stabilisation in the previous stage is secured.

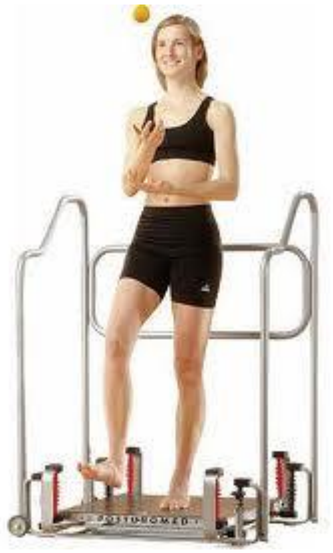

8.1.2 Control Intervention

Added to conventional therapy, as administered to all participants, the control group will receive added LIT. The control intervention will consist of 10 minutes of low intensity endurance training (Borg scale 6-9). The patient will be instructed in treadmill, stationary cycling or cross-trainer functions. The speed should be adjusted to the level where the patient would still be able to talk comfortably.

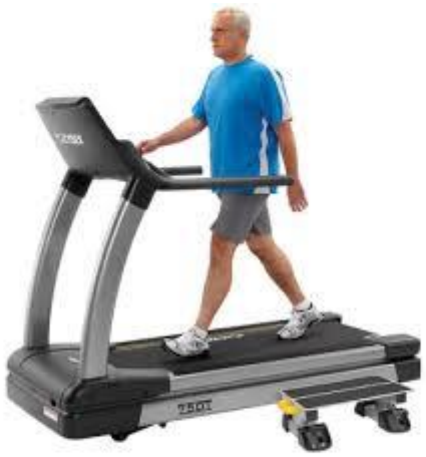

8.1.3 Packaging, Labelling and Supply (re-supply)

Not applicable.

8.1.4 Storage Conditions

Devices are re-usable and do not require any specific storage regulation.

8.2 Administration of experimental and control interventions

8.2.1 Conventional Therapy (both groups)

All participating patients will attend usual care according to their treatment plan (taking part in the study does not affect the treatment plan). A physiotherapy referral requests 9 treatments, each of which takes 30 minutes. These will take place twice a week for 4.5 weeks. The study protocol does not dictate the content of the physiotherapy sessions themselves, but detailed documentation of provided treatments is required (therapy documentation sheet). Additionally, the aim of the

physiotherapy sessions should always include reduction of muscular imbalances and optimisation of posture.

8.2.2 Experimental Intervention

The exercise intervention under investigation is performed with a non-invasive therapy product. The patient stands on a platform with minimal sway and tries to stabilise upright posture on one leg. The aim of each exercise is to train inter-spinal muscles and improve sensorimotor control of postural muscles. The training is applied one to two times per week after the patient's regular visit to physiotherapy. The patient is then instructed in the usage of the device by a therapist who is trained in proprioceptive training methods. The exercise is executed in 5 sets of 1.5 minutes with a 30 to 60 seconds break in-between each set. In order to provoke any training effects, the difficulty of the exercise must be adapted to the individual's skills and abilities. A therapist will be nearby to instruct each stage of difficulty and to promote the patient to the next higher stage once the previous one is mastered adequately. The criteria are defined in the instruction booklet [8].

8.2.3 Control Intervention

The low intensity cardiovascular exercises will be performed a cardio-device of choice. The patients will be instructed to walk (not run) or cycle at a comfortable pace with low intensity for ten minutes. Initial settings and a general introduction will be given by the therapist. Once individual comfort zone (speed, at which participant feels comfortable to walk and talk for 10 minutes) is set, the intensity is not changed.

8.3 Dose / Device modifications

Frequency and dose remain constant over the entire trial. Difficulty levels of the experimental intervention may be adapted as described above.

8.4 Compliance with study intervention

Generally, the patients must conduct the training themselves. A medical fitness instructor will be nearby to oversee the training and will be instructed to record any deviation from the prescribed exercise regimen. The patient will also be asked to record deviations from training regimen on his or her exercise diary. Home exercises will not be controlled for compliance but noted on the diary.

8.5 Data Collection and Follow-up for withdrawn participants

Withdrawn patients will be invited to take part at the assessments only. If refused, intention-to-treat analysis will be applied with carry-forward data.

8.6 Trial specific preventive measures

Not applicable.

8.7 Concomitant Interventions (treatments)

Co-interventions cannot be controlled in the clinical setting of rehabilitation.

8.8 Study Drug / Medical Device Accountability

Not applicable.

8.9 Return or Destruction of Study Drug / Medical Device

Not applicable.

9. STUDY ASSESSMENTS

Table 2: Study procedure chart

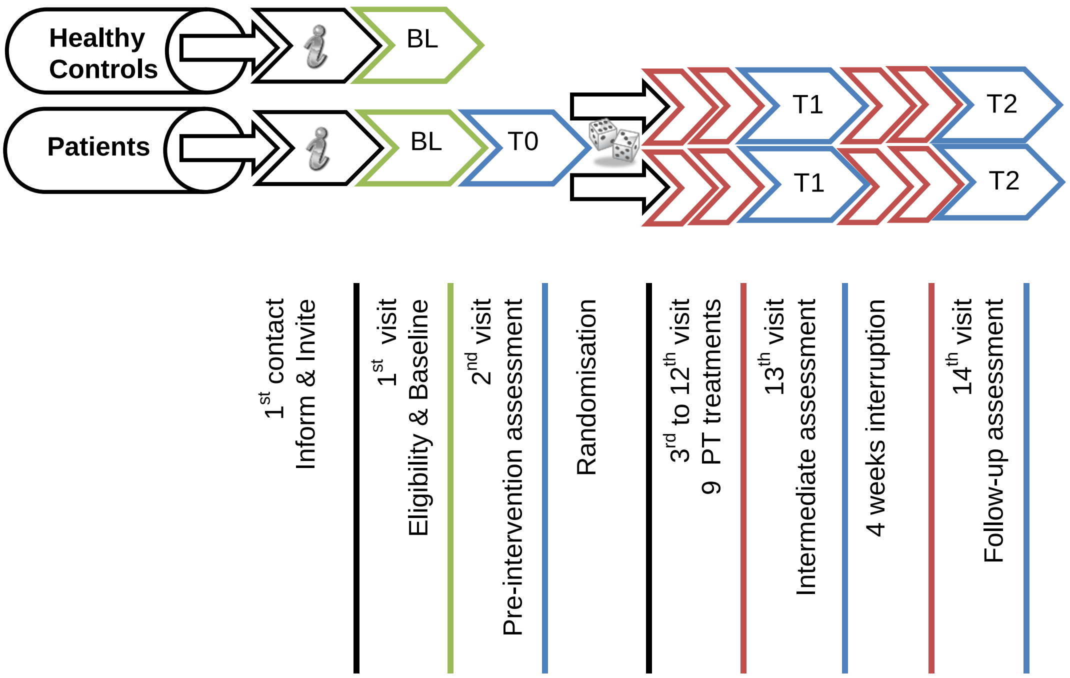

Table 3: Study procedures at each visit:

1st contact: Inform & Invite	If the willingness to partake is confirmed (within 1-2 days), an appointment for eligibility evaluation and baseline assessment is made.
1st visit<br><br>Eligibility & Baseline	4 to 7 days prior to the 2nd visit, a baseline assessment shall confirm eligibility and pre-test data is recorded.<br><br>• Eligibility criteria assessed (incl. medical examination)<br>• Pain & Function assessed with validated questionnaires<br>• Cervical proprioception recorded as cervical joint repositioning sense<br>• Postural control assessed with 2D-motion tracking and UCM analysis
2nd visit<br><br>Pre-intervention assessment	On the day of the first treatment the pre-intervention assessment is recorded.<br><br>• Pain & Function assessed with validated questionnaires<br>• Postural control assessed with 2D-motion tracking and UCM analysis<br>• Cervical proprioception recorded as cervical joint repositioning sense
Randomisation	The patient is allocated to either the experimental or the control group.
3rd to 12th visit<br><br>9 PT treatments	The patient visits the clinic as he or she would have done for normal physiotherapy treatment. Added to the 30 minutes of physiotherapy, the patient attends additional 15 minutes of either PPT or TrT.
13th visit<br><br>Intermediate assessment	1 to 2 days after last treatment, the patient will undergo an intermediate effect assessment.<br><br>• Pain & Function assessed with validated questionnaires<br>• Cervical proprioception recorded as cervical joint repositioning sense<br>• Postural control assessed with 2D-motion tracking and UCM analysis

4 week interruption	
14th visit	1 to 2 days after treatment, the patient will undergo post-intervention effects assessment.<br><br>• Pain & Function assessed with validated questionnaires<br>• Cervical proprioception recorded as cervical joint repositioning sense<br>• Postural control assessed with 2D-motion tracking and UCM analysis

9.1 Experimental procedure

Measurements will take place within the clinic's facilities (Reha Rheinfelden) featuring all necessary instruments to record data, including the Posturomed® to induce instability, the Haider Bioswing accelerometer to record plate deflection, a Zebris force sensor plate to record centre of pressure (COP), high-speed cameras for motion tracking and the motion analysis system (software and computer).

9.2 Assessments of outcomes

9.2.1 Assessment of primary outcome

Whilst preparing the setup and software configuration, the subject will be given 10 minutes time to fill in the personal data sheet, including the VAS scales for pain, and the ODI questionnaire for functional status. The questionnaires will be filled in by the patients at every measurement event (BL, T0, T_1, and T_2).

Self-reported impairment in daily activities will be assessed using the German version of the Oswestry Disability Index (ODI-D), which shows a good reliability. The ODI has ten sections about daily activities (pain, body hygiene, lifting objects, walking, sitting, standing, sleeping, sexual behaviour, social life, and travelling). Each section can be rated from 0 (no pain during activity or pain getting worse) to 5 (I cannot do it myself). The total score is in percentage of the total 50 points, if each section was answered (45, if 9 sections were answered, and so on). The end score will be from 0% (minimal impairment) to 100% (bedridden). A change of 4 points is considered as clinically relevant [26].

Self-reported pain will be assessed using the German version of the Visual Analogue Scale (VAS). It shows a good reliability with a correlation of 0.937 between the first and the second assessment. The VAS is a 100mm line with two endpoints representing the extreme states "no pain" and "pain as bad as it could be". A minimum of 13 mm difference on the scale is considered as clinical relevant [26].

9.2.2 Assessment of secondary outcomes

The secondary outcomes, joint repositioning sense and postural control, will be measured at each measurement event (BL, T0, T_1, and T_2).

The experimental set-up consists of the neuro-orthopaedic therapy system Posturomed® 202 with handrail (Haider Bioswing, Pullenreuth, Germany), an attached provocation module (Haider Bioswing GmbH, Pullenreuth, Germany), a high-speed camera Basler A602F (Basler AG, Ahrensburg, Germany), a personal computer with Windows 8 operating system to which the camera is attached with USB 3.0, an accelerometer attached to the base-plate of the Posturomed®, and optical markers to measure different joint deflections and joint motion. The markers will be tracked with the motion analysis software Templo (v.7.1.304, Contemplas GmbH, Kempten, Germany). COP is measured by means of a Zebris FDM-S force measuring plate on top of the swaying platform.

After evaluating primary outcomes, the participants will be equipped with markers as shown in Figure 2 below. After familiarisation with the Posturomed's labile surface, he or she will be instructed to adopt an upright posture with the arms folded across the chest (in order to keep hip markers visible). On the cue "ready-steady-go", the assessor will release the platform from its 5 cm deflection. The swaying is recorded until complete damping of the oscillation has occurred (approximately 15

seconds). As participants will be standing on both feet, any facilitation of the task through engaging brakes would make the task too easy (as tested in pre-pilot tests). Hence, all of the device's brakes will be released for this test allowing maximal sway. The camera setup will record the entire task. The task will be repeated five times for each patient. The arithmetic mean of the five trials will be used for further analysis.

Optical markers in combination with a high-speed camera are used in the study for motion tracking of joint configurations. The reflecting markers, eleven in total, are placed on the participants as depicted in Figure 2 below: At the corner of the eye, the mastoid process, shoulder (acromion), hip (greater trochanter), knee (lateral femoral condyle), ankle (lateral malleolus), toe, heel and the platform surface. There are additionally two markers on the iliacus superior for calculation of the pelvic tilt angle and one on the floor for calibration purposes. The marker configuration is based on recommendations from Scholz et al. [27]. The markers are 16mm in diameter and will be attached using skin-friendly adhesive tape.

Two cameras will track these reflecting markers with a spatial resolution of 1.5 megapixels and a temporal resolution of 60 frames per second, which has shown to be enough for the purposes (tested in pre-pilot studies). Calibrations will be carried out at the beginning of the performance of the task.

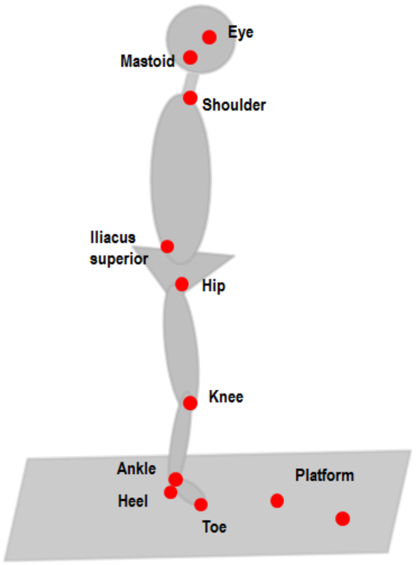

Figure 2: Marker configuration for video-based postural control assessment

An additional setup is installed to measure cervical proprioception (Figure 3). On a wall facing a chair, there are vertical lines that represent the 30° and 60° angle to the left and the right side. Participants are blindfolded to avoid opening of the eyes and visual input during testing. Additionally, they wear a headgear to prevent acoustical input like computer ventilation (instructions are still perceptible). A laser pointer is fixed on the headgear. A computer mouse fixed to the hander side of the participant can be clicked to indicate when the head position is perceived to be at the instructed angle. Each click records the exact time and allows post-measurement synchronisation with the motion-tracking data. Cameras stand both lateral and posterior to the participant. The lateral camera records any possible deviations in posture of the trunk during measurements.

The testing order is randomized. The instructor gives cues following the test protocol. Five trials are used because more than three repetitions seem to be needed in order to reduce variability of the results but less than ten to avoid fatigue and noncompliance of Participants. Participants hold the laser pointer on the target angle line for 15 s (with one click on the left mouse-button for data of reference position). After returning to neutral position, participants replicate the target angle position followed by a mouse-click to confirm position. This procedure is repeated five times for one target angle before proceeding to the next angle. To measure repositioning of neutral position, participants hold in their personal, most natural way of NP and click the mouse. After holding NP for 15s, they perform a full right and left rotation of the head, directly return to NP and click the mouse.

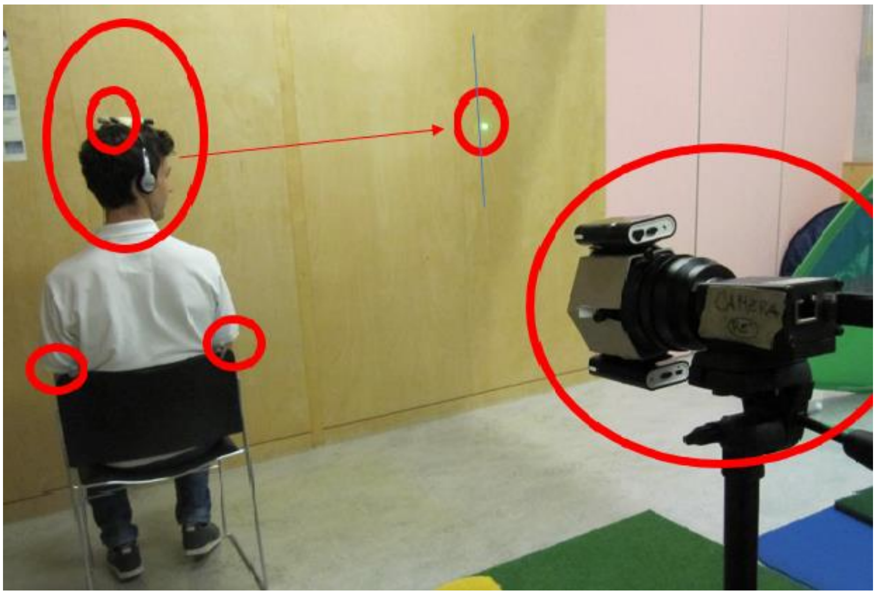

Figure 3: Measurement set-up for cervical proprioception (head-repositioning error).

The setup was pilot-tested in order to define optimal settings for the recording (e.g. light, camera distance, marker-repositioning).

The information on change of angular configuration during the task will be used to apply the uncontrolled manifold approach (UCM). The UCM hypothesis states that the central nervous system (CNS) does not eliminate redundant degrees of freedom. It uses these degrees of freedom to ensure stable performance of motor tasks. A UCM spans a subspace consisting of all joint angles which ensure a stable movement and do not affect the controlled variable. Joint angle combinations which lie in a subspace orthogonal to the UCM lead to a deviation from this stable condition and therefore affect the controlled variable. The mathematical method is described in detail by Scholz et al. [20].

Additionally the joint repositioning error will be analysed from this data. The joint repositioning error is the deflection of a joint or joint angle after a movement in comparison to the joint configuration before the movement. The three-point angle on all recorded segments will be analysed. This error can be a measure for conscious body position perception [28].

There is a pressure sensor (Zebris FDM-S) and an accelerometer with a sampling frequency of 1000Hz on the base-plate, which will be used for calculation of the centre of pressure and the sway of the platform, respectively. These parameters provide further information about postural control.

9.2.3 Assessment of other outcomes of interest

At BL socio-demographic data will be recorded to characterise the included patients (age, gender, activity level, and profession.

9.2.4 Assessment of safety outcomes

Any unexpected occurrences will be recorded to the case report form.

9.2.4.1 Adverse events

Adverse events will be recorded to the CRF and transferred to a central documentation sheet.

9.2.4.2 Laboratory parameters

Not applicable.

9.2.4.3 Vital signs

Not applicable.

9.2.5 Assessments in participants who prematurely stop the study

Not applicable.

9.3 Procedures at each visit

Please refer to Table 3.

10. SAFETY

10.1 Drug studies

Not applicable.

10.2 Medical Device Category C studies

Not applicable.

10.3 Medical Device Category A studies

10.3.1 Definition and Assessment of safety related events

In our study an adverse event will be defined as any untoward medical occurrence in a subject without regard to the possibility of a causal relationship.

10.3.2 Reporting of Safety related events

Adverse events will be collected after the participant has provided consent and enrolled in the study. If a participant experiences an adverse event after the informed consent document is signed (entry) but he or she has not started to receive study intervention, the event will be reported as not related to study intervention. All adverse events occurring after entry into the study and until discharge will be recorded. An adverse event that meets the criteria for a serious adverse event (SAE) between study enrolment and discharge will be reported to the CEC as an SAE. If exercise is discontinued as a result of an adverse event, study personnel will document the circumstances and data leading to discontinuation of treatment.

A serious adverse event for this study is any untoward medical occurrence that is believed by the investigators to be causally related to exercise and results in any of the following: Life-threatening condition (that is, immediate risk of death); severe or permanent disability, prolonged hospitalization, or a significant hazard as determined by the treating therapists. Serious adverse events occurring after a participant is discontinued from the study will not be reported.

11. STATISTICAL METHODS

11.1 Hypothesis

1° Null Hypothesis:	Functional status and self-reported pain will reduce significantly in both groups, CG and EG, but no group interaction is expected.
1° Alternative Hypothesis:	EG will show significantly more improvement in all primary and secondary outcomes as compared to CG.
2° Null Hypothesis:	Compared to healthy controls, patients with CNLBP will show no difference in postural control with optimal joint configuration variability when adjusting COM after destabilisation as compared to healthy controls.
2° Alternative Hypothesis:	Patients with CNLBP will show different postural variability to adjust to destabilisation as compared to healthy controls.
3° Null Hypothesis:	Compared to healthy controls, patients with CNLBP will show no difference in cervical proprioception acuity as compared to healthy controls.
3° Alternative Hypothesis:	Patients with CNLBP will show reduced cervical proprioception acuity as compared to healthy controls.

11.2 Determination of Sample Size

This is the first study of its kind to investigate the effects of a sensorimotor training on dynamic postural control in patients with CNLBP and is therefore of explorative nature. Related to the primary hypothesis, however, a study using the same outcomes investigated the effects of treadmill training on gait in down-syndrome children and compared them to healthy subjects [29]. Although this is not a comparable study population it was used to estimate the power of the test. With a mean between-group difference of 1.4 and a pooled standard deviation of 0.19 the study revealed highly significant group effects (N=15, p<0.01).

Several studies have investigated the effects of sensorimotor training on pain and functional status. Two of the most recent ones [30, 31] have found significant time and group interactions using the same outcomes. Applying the results of these studies to a sample size calculation with an alpha value of 5% and the desired power 80%, it is expected that 10 patients per arm would suffice to reveal group differences and to detect change.

In terms of the secondary hypothesis, it must be referred to an article which used a similar motor task in an experimental setup with cross-sectional analysis of healthy participants. For the detection of within-subject difference of control parameters of the uncontrolled manifold approach, Scholz et al. [27] found that with a deflection of 0.06 m, the projections of joint configurations to the manifold differed significantly to the projection in the range space (N=9, $F_{1,8}$=25.8, p<0.001). With a mean difference of 0.055 and a standard deviation of 0.015, this calculates to a power of >0.9.

Considering these findings and taking into account the explorative approach of this trial, a total number of 30 participants is planned (10 healthy subjects and 10 patients per intervention arm).

11.3 Statistical criteria of termination of trial

As this is an explorative study there are no statistical criteria that would lead to termination of the trial. In terms of quantitative analysis, the results will be reported in any case, irrespective of the achieved sample size at the end of the trial duration.

11.4 Planned Analyses

Baseline imbalance between groups will be tested with a student t-test. Differences in means between healthy participants and patients with CNLBP will be analysed with independent student t-tests. A mixed (2) Group x (2) ME ANOVA with repeated measures during ME will be conducted for each dependent variable individually (Pain VAS, Functional Status ODI, Postural Control COM and CM, Proprioception JRE, and Motor Equivalence UCM).

11.4.1 Datasets to be analysed, analysis populations

All participants will be analysed, intention-to-treat analysis will be applied. No sub-group analysis is intended.

11.4.2 Primary Analysis

Primary analysis will be done by the PL at conclusion of all assessments. The PL will remain uninformed about group allocation until completion of analysis.

11.4.3 Secondary Analyses

No secondary analysis is planned.

11.4.4 Interim analyses

No interim analysis is planned.

11.4.5 Safety analysis

Safety parameters will be monitored by the sponsor-investigator and the PL. At the end of the trial the quantities and natures of any adverse events will be summarised for the report.

11.4.6 Deviation(s) from the original statistical plan

No deviations are intended.

11.5 Handling of missing data and drop-outs

Missing data will be treated with last observation carried forward.

12. QUALITY ASSURANCE AND CONTROL

12.1 Data handling and record keeping / archiving

All study-related information will be stored securely at research department of the Reha Rheinfelden. Participant information will be stored in locked file cabinets in areas with limited access. All reports, data collection, process, and administrative forms will be identified by a coded ID number only to maintain participant confidentiality. All records that contain names or other personal identifiers, such as locator forms and informed consent forms, will be stored separately from study records identified by code number. All local databases will be secured with password-protected access systems. Forms, appointment books, and any other listings that link participant ID numbers to other identifying information will be stored in a separate, locked file in an area with limited access.

12.1.1 Case Report Forms

Questionnaires will be filled in by patients. The final scores (i.e. VAS and ODI score) will be recorded to the paper-CRF (p-CRF) by the PL or a study assistant. Secondary outcomes, i.e. motion tracking data and platform oscillation, will be recorded electronically. The calculated outcomes for postural control (i.e. averaged joint angle and UCM-Index) will be transferred to the p-CRF by the PL and a study assistant (double data entry).

12.1.2 Specification of source documents

Questionnaires scores will be transferred from the paper questionnaires (VAS and ODI) which will be filled in by the patients.

Dose and frequency (i.e. compliance) will be derived from the paper exercise diary filled in by the patients.

Therapy documentation for the conventional therapy is recorded on a pre-defined documentation sheet designed to record mode and usage of additional devices.

12.1.3 Record keeping / archiving

All study data will be archived for 10 years after study termination or premature termination of the clinical trial. All digital data is archived on the clinic’s server and backed up daily. Video-data is stored on external hard-drives to spare server capacities. Paper data is archived in the research department’s office, which is not accessible to the public.

12.2 Data management

All study related patient data will be entered directly into the digitalised and anonymised case report forms. Results from the written questionnaires will be transferred immediately. Recorded joint configurations are stored as raw data to compute the UCM and CM in a separate step.

Therapy documentation and training protocols will be transferred to an anonymised log-book using study identification numbers for recognition. No personal data, that could identify the patients, will be stored for study purposes or used for analyses.

12.2.1 Data Management System

A VMWAre Horizon environment is maintained in the clinic which is accessed via the local thin client. It runs Windows 7 platform and provides the MS Office package needed for the report. However, statistical and motion tracking software is not installed on the clinic’s server and must be operated with an external notebook provided by the ETH Zurich. All raw data is recorded straight to the notebook and backed up on the server (only anonymised motion tracking data, i.e. angular variation and postural information). Video files are stored on the Notebook and the external hard drive, but not on the Server.

Data structures will be organised using Matlab (MATLAB 2014a., The MathWorks Inc.: Natick, Massachusetts).

12.2.2 Data security, access and back-up

The digital data is backed up daily on the clinic's server (except Video files, these are backed up on a second external hard drive once a month).

12.2.3 Analysis and archiving

A data structure in Matlab will contain an array for each patient (ID.01 to ID.20). Each array will contain vector information of the assessed outcomes. In a second step, a technical assistant, not involved in the study, will allocate a new identification number to each participant and separate the control from the experimental group. This new data sheet will then be handed back to the PL who will conduct final analysis. This method reduces the risk of bias as the PL remains blinded to each individual's group allocation during statistical analysis. Specifically, at the end of the study, the folder containing ID01 to ID20 data is edited by the technical assistant to re-randomise all ID numbers and transform these to any number between 87300 and 87390 with either an A or B as suffix, depending on the group, e.g. 87345_A. The PL will not know whether A or B is the experimental group until analysis is completed.

Study related original patient documents will be archived for 10 years on an external hard-drive. After that period patient documents will be destroyed in accordance with the clinic's data destruction guidelines.

Non-numeric data is entered onto excel sheets and coded. The coded lists are also exported to Matlab and allocated to the patient within the data structure.

12.2.4 Electronic and central data validation

Electronic data is verified by cross-checking of entered data by other members of the research group. Each file and case report form can only be saved when all relevant fields are filled in and analysis completed. A warning will appear when data is missing or data seems out range or impossible (e.g compared to the rest of the group or larger than maximum, etc.).

12.3 Monitoring

The source data and documents will made accessible to monitors and questions are answered during monitoring. Monitoring will be organised internally by members of the research group who will cross-check case report forms and control file structures, folder contents and informed consent.

12.4 Audits and Inspections

No additional auditing is intended for the study. Study documentation and the source data/documents are accessible to auditors and inspectors from local authorities, e.g. from the ethical committee. Questions are answered during inspections. All involved parties must keep the participant data strictly confidential.

12.5 Confidentiality, Data Protection

Direct access to source documents will be permitted for purposes of monitoring (12.3), audits and inspections (12.4) (ICHE6, 6.10). For external audits, monitoring events, and inspections authorised personnel (ethics committee, CTI representatives) will have direct access to all study data. Patient data will be treated as strictly confidential and all documents will remain in the secured and locked office of the trial centre.

12.6 Storage of biological material and related health data

Not applicable.

13. PUBLICATION AND DISSEMINATION POLICY

The project shall eventually result in three to four publications including the findings of the cross-sectional study, a retest analysis and the RCT. Publication of a technical paper on the methodologies will be considered. It is intended to publish in open access journals relevant to the field. Authorship has been negotiated within the steering committee.

14. FUNDING AND SUPPORT

14.1 Funding

No funding. The project is part of the PL's Ph.D. which is financed by the sponsor-investigator. Personal costs of the PL, the therapists etc. are covered by the sponsor-investigator.

14.2 Other Support

The measurement devices are provided by the ETH Zurich, Institute for Human Movement Science.

15. INSURANCE

Insurance will be provided by the Sponsor-Investigator. A copy of the certificate is filed in each investigator site file and the trial master file (National Suisse, policy number 40.025.077)

16. REFERENCES

1. Airaksinen O, Brox JI, Cedraschi C, Hildebrandt J, Klaber-Moffett J, Kovacs F, Mannion AF, Reis S, Staal JB, Ursin H *et al*: **Chapter 4. European guidelines for the management of chronic nonspecific low back pain**. *Eur Spine J* 2006, **15 Suppl 2**:S192-300.
2. Läubli T: **Arbeit und Gesundheit - Erkrankung und Beschwerden des Bewegungsapparates**. In. Edited by SECO SSfEA: State Secretariat for Economic Affairs SECO; 2009.
3. Zusman M: **Belief reinforcement: one reason why costs for low back pain have not decreased**. *Journal of multidisciplinary healthcare* 2013, **6**:197-204.
4. Bekkering GE, Hendriks HJM, Koes BW, Oostendorp RAB, Ostelo RWJG, Thomassen JMC, M. W. vT: **National practice guidelines for physical therapy in patients with low back pain**. In.: KNGF; 2003: 29-29.
5. Kent P, Mjøsund HL, Petersen DHD: **Does targeting manual therapy and/or exercise improve patient outcomes in nonspecific low back pain? A systematic review**. *BMC Medicine* 2010, **8**.
6. Janda V, Frank C, Liebenson C: **Evaluation of Muscular Imbalance**. In: *Rehabilitation of the Spine: A Practitioner's Manual.* edn. Edited by Liebenson C. Baltimore: Lippincott Williams & Wilkins; 2006: 203-225.
7. Page P: **Sensorimotor training: A "global" approach for balance training**. *Journal of Bodywork and Movement Therapies* 2006, **10**(1):77-84.
8. Rasev E: **Therapieanleitung für die posturale Therapie nach Dr. Eugen Rasev**. In. Edited by Bioswing H; 2010.
9. Otte C, Rasev E: **Posturale Aspekte der Schmerztherapie des Bewegungssystems**. *Manuelle Medizin - Springer Verlag* 2010, **48**:267-274.
10. Andersen JH, Haahr JP, Frost P: **Risk factors for more severe regional musculoskeletal symptoms: a two-year prospective study of a general working population**. *Arthritis Rheum* 2007, **56**:1355-1364.
11. Macfarlane GJ, Thomas E, Papageorgiou AC, Croft PR, Jayson MI, Silman AJ: **Employment and physical work activities as predictors of future low back pain**. *Spine (Phila Pa 1976)* 1997, **22**:1143-1149.
12. Roelen CA, Schreuder KJ, Koopmans PC, Groothoff JW: **Perceived job demands relate to self-reported health complaints**. *Occup Med (Lond)* 2008, **58**:58-63.
13. Horak FB: **Postural orientation and equilibrium: what do we need to know about neural control of balance to prevent falls?** *Age and ageing* 2006, **35 Suppl 2**:ii7-ii11.
14. Nordin M, Carragee EJ, Hogg-Johnson S, Weiner SS, Hurwitz EL, Peloso PM, Guzman J, van der Velde G, Carroll LJ, Holm LW *et al*: **Assessment of neck pain and its associated disorders: results of the Bone and Joint Decade 2000-2010 Task Force on Neck Pain and Its Associated Disorders**. *Spine (Phila Pa 1976)* 2008, **33**(4 Suppl):S101-122.
15. Hodges PW: **Pain and motor control: From the laboratory to rehabilitation**. *Journal of Electromyography and Kinesiology* 2011, **21**:220-228.
16. Bergmark A: **Stability of the lumbar spine. A study in mechanical engineering**. *Acta orthopaedica Scandinavica Supplementum* 1989, **230**:1-54.
17. Comerford MJ, Mottram SL: **Movement and stability dysfunction--contemporary developments**. *Man Ther* 2001, **6**(1):15-26.
18. Ashton-Miller JA, Wojtys EM, Huston LJ, Fry-Welch D: **Can proprioception really be improved by exercises?** *Knee Surg Sports Traumatol Arthrosc* 2001, **9**(3):128-136.
19. Kim D, Van Ryssegem G, Hong J: **Overcoming the Myth of Proprioceptive Training**. *Clinical Kinesiology (Spring)* 2011, **65**(1):18-28.
20. Scholz JP, Schoner G: **The uncontrolled manifold concept: identifying control variables for a functional task**. *Exp Brain Res* 1999, **126**(3):289-306.
21. Harbourne RT, Stergiou N: **Movement variability and the use of nonlinear tools: principles to guide physical therapist practice**. *Phys Ther* 2009, **89**(3):267-282.

22. Boeer J, Mueller O, Krauss I, Haupt G, Horstmann T: **[Reliability of a measurement technique to characterise standing properties and to quantify balance capabilities of healthy subjects on an unstable oscillatory platform (Posturomed)]**. *Sportverletz Sportschaden* 2010, **24**:40-45.

23. Muller O, Gunther M, Krauss I, Horstmann T: **[Physical characterization of the therapeutic device Posturomed as a measuring device--presentation of a procedure to characterize balancing ability]**. *Biomed Tech (Berl)* 2004, **49**:56-60.

24. Ganz P, Winterfeld S: **Evaluation der posturalen Therapie nach Dr. Rasev bei chronischen, unspezifischen Rückenschmerzen**. Nijmegen: Hogeschool van Arnhem; 2009.

25. McCaskey MA, Schuster-Amft C, Wirth B, de Bruin ED: **Effects of proprioceptive exercises on pain and function in chronic neck- and low back pain rehabilitation: A systematic literature review**. *BMC Musculoskelet Disord* 2014, **Under Review**.

26. Oesch P: **Assessments in der muskuloskelettalen Rehabilitation**. Bern: Huber-Hans Verlag; 2007.

27. Scholz JP, Schoner G, Hsu WL, Jeka JJ, Horak F, Martin V: **Motor equivalent control of the center of mass in response to support surface perturbations**. *Exp Brain Res* 2007, **180**(1):163-179.

28. Riemann BL, Myers JB, Lephart SM: **Sensorimotor system measurement techniques**. *Journal of athletic training* 2002, **37**(1):85-98.

29. Chung SH, Lee JS, Yoon JS: **Effects of stabilization exercise using a ball on mutifidus cross-sectional area in patients with chronic low back pain**. *Journal of Sports Science and Medicine* 2013, **12**(3):533-541.

30. Beinert K, Taube W: **The Effect of Balance Training on Cervical Sensorimotor Function and Neck Pain**. *J Motor Behav* 2013, **45**(3):271-278.

31. Lee HI, Song J, Lee HS, Kang JY, Kim M, Ryu JS: **Association between Cross-sectional Areas of Lumbar Muscles on Magnetic Resonance Imaging and Chronicity of Low Back Pain**. *Annals of Rehabilitation Medicine* 2011, **35**(6):852-852.
